# Recreation of the periodic table with an unsupervised machine learning algorithm

**DOI:** 10.1038/s41598-021-81850-z

**Published:** 2021-02-26

**Authors:** Minoru Kusaba, Chang Liu, Yukinori Koyama, Kiyoyuki Terakura, Ryo Yoshida

**Affiliations:** 1 The Graduate University for Advanced Studies, SOKENDAI, Tachikawa, Tokyo 190-8562 Japan; 2 The Institute of Statistical Mathematics, Research Organization of Information and Systems, Tachikawa, Tokyo 190-8562 Japan; 3 National Institute for Materials Science, Tsukuba, Ibaraki 305-0047 Japan; 4 National Institute of Advanced Industrial Science and Technology, Tsukuba, Ibaraki 305-8560 Japan

**Keywords:** Cheminformatics, Materials science, Statistics

## Abstract

In 1869, the first draft of the periodic table was published by Russian chemist Dmitri Mendeleev. In terms of data science, his achievement can be viewed as a successful example of feature embedding based on human cognition: chemical properties of all known elements at that time were compressed onto the two-dimensional grid system for a tabular display. In this study, we seek to answer the question of whether machine learning can reproduce or recreate the periodic table by using observed physicochemical properties of the elements. To achieve this goal, we developed a periodic table generator (PTG). The PTG is an unsupervised machine learning algorithm based on the generative topographic mapping, which can automate the translation of high-dimensional data into a tabular form with varying layouts on-demand. The PTG autonomously produced various arrangements of chemical symbols, which organized a two-dimensional array such as Mendeleev’s periodic table or three-dimensional spiral table according to the underlying periodicity in the given data. We further showed what the PTG learned from the element data and how the element features, such as melting point and electronegativity, are compressed to the lower-dimensional latent spaces.

## Introduction

The periodic table is a tabular arrangement of elements such that the periodic patterns of their physical and chemical properties are clearly understood. The prototype of the current periodic table was first presented by Mendeleev in 1869^1^. At that time, about 60 elements and their few chemical properties were known. When the elements were arranged according to their atomic weight, Mendeleev noticed an apparent periodicity and an increasing regularity. Inspired by this discovery, he constructed the first periodic table. Despite the subsequent emergence of significant discoveries^2,3^, including the modern quantum mechanical theory of the atomic structure, Mendeleev’s achievement is still the de facto standard. Regardless, the design of the periodic table continues to evolve, and hundreds of periodic tables have been proposed in the last 150 years^4,5^. The structures of these proposed tables have not been limited to the two-dimensional tabular form, but also spiral, loop, or three-dimensional pyramid forms^6–8^.

The periodic tables proposed so far have been products of human intelligence. However, a recent study has attempted to redesign the periodic table using computer intelligence—machine learning^9^. From this approach, building a periodic table can be viewed as an unsupervised learning task. Precisely, the observed physicochemical properties of elements are mapped onto regular grid points in a two-dimensional latent space such that the configured chemical symbols adequately capture the underlying periodicity and similarity of the elements. Lemes and Pino^9^ used Kohonen’s self-organizing map (SOM)^10^ to place five-dimensional features of elements (i.e. atomic weight, radius of connection, atomic radius, melting point, and reaction with oxygen) into two-dimensional rectangular grids. This method successfully placed similarly behaved elements into neighbouring sub-regions in the lower-dimensional spaces. However, the machine learning algorithms never reached Mendeleev’s achievement as they missed important features such as between-group and between-family similarities.

In this study, we created various periodic tables using a machine learning algorithm. The dataset that we used consisted of 39 features (melting points, electronegativity, and so on) of 54 elements with the atomic number 1–54, corresponding to hydrogen to xenon (Fig. S1 for the heatmap display). A wide variety of dimensionality reduction methods has so far been made available, such as principal component analysis (PCA), kernel PCA^11^, isometric feature mapping (ISOMAP)^12^, local linear embedding (LLE)^13^, and t-distributed stochastic neighbour embedding (t-SNE)^14^. However, none of these methods could well visualize underlying periodic laws (Supplementary Fig. S3). To begin with, none of these methods offers a tabular representation. The task of building a periodic table can be regarded as the dimension reduction of the element data to arbitrary given ‘discrete’ points rather than a continuous space. To the best of our knowledge, no existing framework is available for such table summarization tasks. Therefore, we developed a new unsupervised machine learning algorithm called the periodic table generator (PTG), which relies on the generative topographic mapping (GTM)^15^ with latent variable dependent length-scale and variance (GTM-LDLV)^16^. One of the advantages of using the GTM-LDLV arises from its ability to represent complex response surfaces. Elemental data shows a complex response surface on the feature space. Controlling the two hyperparameters, the GTM-LDLV can flexibly represent functions whose smoothness and amplitude vary locally in the feature space. With this model, we automate the process of translating patterns of high-dimensional feature vectors to an arbitrary given layout of lower dimensional point clouds.

The PTG produced various arrangements of chemical symbols, which organized, for example, a two-dimensional array such as Mendeleev’s table or three-dimensional spiral table according to the underlying periodicity in the given data. We will show what the machine intelligence learned from the given data and how the element features were compressed to the reduced dimensionality representations. The periodic tables can also be regarded as the most primitive descriptor of chemical elements. Hence, we will highlight the representation capability of such element-level descriptors in the description of materials that were used in machine learning tasks of materials property prediction.

## Materials and methods

### Computational workflow

The workflow of the PTG begins by specifying a set of point clouds, called ‘nodes’ hereafter, in a low-dimensional latent space to which chemical elements with observed physicochemical features are assigned. The nodes can take any positional structure such as equally spaced grid points on a rectangular for an ordinal table, spiral, cuboid, cylinder, cone, and so on. A Gaussian process (GP) model^17^ is used to map the pre-defined nodes to the higher-dimensional feature space in which the element data are distributed. A trained GP defines a manifold in the feature space to be fitted with respect to the observed element data. The smoothness of the manifold is governed by a specified covariance function called the kernel function, which associates the similarity of nodes in the latent space with that in the feature space. The estimated GP defines a posterior probability or responsibility of each chemical element belonging to one of the nodes. An element is assigned to one node with the highest posterior probability.

**Figure 1:**
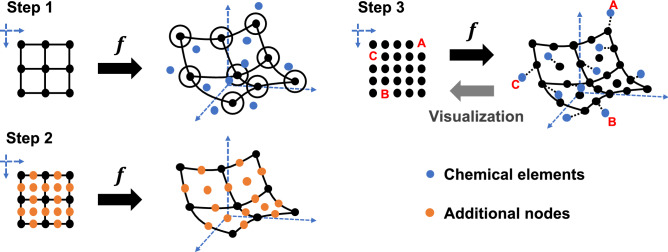
Workflow of PTG that relies on a three-step coarse-to-fine strategy to reduce the occurrence of undesirable matching between chemical elements and redundant nodes.

As indicated by the failure of some existing methods of statistical dimension reduction, such as PCA, t-SNE, and LLE, the manifold surface of the mapping from chemical elements to their physiochemical properties is highly complex. Therefore, we adopted the GTM-LDLV as a model of PTG, which is a GTM that can model locally varying smoothness in the manifold. To ensure non-overlapping assignments such that no multiple elements shared the same node, we operated the GTM-LDLV with the constraint of one-to-one matching between nodes and elements. To satisfy this, the number of nodes, $K$, has to be larger than the number of elements, $N$. However, a direct learning with $K>N$ suffers from high computational costs and instability of the estimation performance. Specifically, the use of redundant nodes leads to many suboptimal solutions corresponding to undesirable matchings to the chemical elements. To alleviate this problem, the PTG was designed to take a three-step procedure (Fig. 1) that relies on a coarse-to-fine strategy. In the first step, we operated the training of GTM-LDLV with a small set of nodes such that $K<N$. In the following step, we generated additional nodes such that $K>N$, and the expanded node-set was transferred to the feature space by performing the interpolative prediction made by the given GTM-LDLV. Finally, the pre-trained model was fine-tuned subject to the one-to-one matching between the $N$ elements and the $K$ nodes for tabular construction. The procedure for each step is detailed below.

*Step 1* (GTM-LDLV): the first step of the PTG is the same as the original GTM-LDLV. In the GTM-LDLV, $K$ nodes, ${{\varvec{u}}}_{1}, \dots , {{\varvec{u}}}_{K}$, arbitrarily arranged in the $L$-dimensional latent space are first prepared. Then we build a nonlinear function ${\varvec{f}}({{\varvec{u}}}_{k})$ that maps the pre-defined nodes to the $D$-dimensional feature space. The model ${\varvec{f}}({{\varvec{u}}}_{k})$ defines an $L$-dimensional manifold in the $D$-dimensional feature space, which is fitted with respect the $N$ data points of element features. The dimension of the latent space is set to $L\le 3$ for visualization.

It is assumed that the $D$-dimensional feature vector ${{\varvec{x}}}_{n}$ of element $n$ is generated independently from a mixture of *K* Gaussian distributions, where the mixing rates are all equal to $1/K$, and the mean and the covariance matrix of each distribution are ${{\varvec{y}}}_{k}={\varvec{f}}\left({{\varvec{u}}}_{k}\right)$ and ${\beta }^{-1}{\varvec{I}}$, respectively (${\varvec{I}}$ denotes the identity matrix). According to the GTM-LDLV, the mean ${\varvec{f}}({{\varvec{u}}}_{k})$ is modelled to be the product of two functions, a $D$-dimensional vector-valued function ${\varvec{h}}({{\varvec{u}}}_{k})$ and a positive scalar function $g({{\varvec{u}}}_{k})$. Here, we introduce a vector of $K$ latent variables, ${{\varvec{z}}}_{n}={({z}_{1n},\dots ,{z}_{Kn})}^{^{\prime}}$, that indicates the assignment of element $n$ to one of the given $K$ nodes. The $k$th entry ${z}_{kn}$ takes the value of 1 if ${{\varvec{x}}}_{n}$ is generated by the $k$th component distribution, and 0 otherwise. Here, let ${\varvec{X}}$ denote a matrix of ${{\varvec{x}}}_{1}, \dots , {{\varvec{x}}}_{N}$ of the elements, and ${\varvec{Z}}$ be a matrix of ${{\varvec{z}}}_{1}, \dots , {{\varvec{z}}}_{N}$. Then, their joint distribution is given by

1 $$\begin{array}{c}p\left({\varvec{X}},{\varvec{Z}}|{\varvec{g}},{\varvec{H}},\beta \right)={K}^{-N}\prod_{n=1}^{N}\prod_{k=1}^{K}{N\left({{\varvec{x}}}_{n}|{{\varvec{y}}}_{k},{\beta }^{-1}{\varvec{I}} \right)}^{{z}_{kn}},\end{array}$$

2 $$\begin{array}{c}{{\varvec{y}}}_{k}=f\left({{\varvec{u}}}_{k}\right)= g\left({{\varvec{u}}}_{k}\right)h\left({{\varvec{u}}}_{k}\right),\end{array}$$

where $N\left(\cdot |{\varvec{\mu}},{\varvec{\Sigma}}\right)$ denotes the Gaussian density function with mean ${\varvec{\mu}}$ and covariance matrix ${\varvec{\Sigma}}$, ${\varvec{g}}$ is a vector of $g\left({{\varvec{u}}}_{k}\right) \left(k=1,\dots , K\right)$, and ${\varvec{H}}$ is a matrix of ${\varvec{h}}\left({{\varvec{u}}}_{k}\right) \left(k=1,\dots ,K\right)$.

The prior distribution of $g({\varvec{u}})$ is given as a truncated GP with mean 0 and covariance function ${c}_{g}({{\varvec{u}}}_{i},{{\varvec{u}}}_{j};{{\varvec{\xi}}}_{g})$, which handles positive-bounded random functions. The prior distribution of the $d$th entry ${h}_{d}({\varvec{u}})$ of ${\varvec{h}}({\varvec{u}})$ is given as a GP with mean $0$ and covariance function ${c}_{h}({{\varvec{u}}}_{i},{{\varvec{u}}}_{j})$. To be specific, the covariance functions, ${c}_{g}({{\varvec{u}}}_{i},{{\varvec{u}}}_{j};{{\varvec{\xi}}}_{g})$ and ${c}_{h}({{\varvec{u}}}_{i},{{\varvec{u}}}_{j})$, are given by

3 $$\begin{array}{c}{c}_{g}\left({{\varvec{u}}}_{i},{{\varvec{u}}}_{j};{{\varvec{\xi}}}_{g}\right)={\nu }_{g}\bullet {\text{e}}{\text{x}}{\text{p}}\left(-\frac{{\Vert {{\varvec{u}}}_{i}-{{\varvec{u}}}_{j}\Vert }^{2}}{2{l}_{g}}\right),\end{array}$$

4 $$\begin{array}{c}{c}_{h}\left({{\varvec{u}}}_{i},{{\varvec{u}}}_{j}\right)={\left\{\frac{2l\left({{\varvec{u}}}_{i}\right)l\left({{\varvec{u}}}_{j}\right)}{{l}^{2}\left({{\varvec{u}}}_{i}\right)+{l}^{2}\left({{\varvec{u}}}_{j}\right)}\right\}}^\frac{L}{2}{\text{e}}{\text{x}}{\text{p}}\left(-\frac{{\Vert {{\varvec{u}}}_{i}-{{\varvec{u}}}_{j}\Vert }^{2}}{{l}^{2}\left({{\varvec{u}}}_{i}\right)+{l}^{2}\left({{\varvec{u}}}_{j}\right)}\right).\end{array}$$

In Eq. (3), the hyperparameter ${{\varvec{\xi}}}_{g}$ consists of ${\nu }_{g}$ and ${l}_{g}$, referred to as the variance and the length-scale, that control the magnitude of variances and smoothness of a positive-valued function $g({\varvec{u}})$ generated from the GP. In Eq. (4), the length-scale parameter $l\left({\varvec{u}}\right)$ is a function of ${\varvec{u}}$ and parameterized as $l\left({\varvec{u}}\right)={\text{exp}}\left(r\left({\varvec{u}}\right)\right)$ with the function $r({\varvec{u}})$ following the GP with mean 0 and covariance function ${c}_{r}({{\varvec{u}}}_{i},{{\varvec{u}}}_{j};{{\varvec{\xi}}}_{r})$. Finally, a gamma prior is placed on the precision parameter $\beta$ in Eq. (1).

The covariance function in Eq. (4) is the key in the GTM-LDLV. In general, a covariance function in a GP governs a degree of preservation between the similarity of any inputs, e.g. ${{\varvec{u}}}_{i}$ and ${{\varvec{u}}}_{j}$, and the similarity of their outputs. The heterogeneous variance over the latent space in Eq. (3) can bring locally varying smoothness in resulting manifolds in the feature space. In addition, the variance function is statistically estimated with the hierarchically specified GP prior based on the covariance function ${c}_{r}({{\varvec{u}}}_{i},{{\varvec{u}}}_{j};{{\varvec{\xi}}}_{r})$.

The unknown parameter to be estimated is ${\varvec{\theta}}=\left\{{\varvec{Z}},\beta ,{\varvec{g}},{\varvec{H}},{\varvec{r}}\right\}$. In the GTM-LDLV, the posterior distribution $p({\varvec{\theta}}|{\varvec{X}})$ is approximately evaluated using a Markov Chain Monte Carlo (MCMC) method. Iteratively sampling from the full conditional posterior distribution for each $\{{\varvec{Z}},\beta ,{\varvec{g}},{\varvec{H}},{\varvec{r}}\}$, we obtained a set of ensembles that follow the posterior distribution approximately. By taking the ensemble average over the samples from $p({\varvec{\theta}}|{\varvec{X}})$, the parameters of the GTM-LDLV are estimated. A detailed description of the GTM-LDLV is given in the Supplementary Information section.

*Step 2* (Node expansion): to avoid the occurrence of improper assignments of the $N$ elements to a redundant set of nodes, we adopt a coarse-to-fine strategy. Starting from an initially trained GP model of $K<N$ at step 1, we refine the model with an increased number of nodes $K\ge N$. For example, $5\times 5$ nodes evenly arranged on the area $\left[-1, 1\right]\times \left[-1, 1\right]$ at step 1 are incremented to $K=9\times 9$ by placing additional nodes at middle points of the line segments connecting between each node. With the currently given parameters, we can infer the values of $r\left({\varvec{u}}\right)$ of the covariance function in Eq. (4) at the expanded nodes, ${{\varvec{u}}}_{1},\dots , {{\varvec{u}}}_{K}$. Likewise, the values of $g\left({\varvec{u}}\right)$ and ${\varvec{h}}\left({\varvec{u}}\right)$ are interpolated. By performing such initialization, we proceed to the next round of the GTM-LDLV.

*Step 3* (GTM-LDLV subject to one-to-one assignments): finally, the resulting GTM-LDLV is fine-tuned to obtain a tabular display by running the above procedure subject to a one-to-one matching between the $N$ elements and the $K$ nodes. By definition, the conditional posterior distribution of the assignment variables is represented as

$$p\left({\varvec{Z}}|{\varvec{X}},{{\varvec{\theta}}}_{-{\varvec{Z}}}\right)\propto \prod_{n=1}^{N}\prod_{k=1}^{K}{{\text{exp}}\left({-\frac{\beta }{2}\Vert {{\varvec{x}}}_{n}-{{\varvec{y}}}_{k}\Vert }^{2}\right)}^{{z}_{kn}}=\mathrm{exp}\left(-\frac{\beta }{2}{\sum }_{n=1}^{N}{\sum }_{k=1}^{K}{z}_{kn}{\Vert {{\varvec{x}}}_{n}-{{\varvec{y}}}_{k}\Vert }^{2}\right),$$

where ${{\varvec{\theta}}}_{-{\varvec{A}}}$ represents a set of the parameters obtained by removing ${\varvec{A}}$ from ${\varvec{\theta}}$. In the MCMC calculation in step 1, we iteratively draw a sample of ${\varvec{Z}}$ from this distribution. Here, instead of performing the random sampling, we conduct the maximization of the logarithmic posterior with respect to ${\varvec{Z}}$ subject to the constraint of one-to-one assignments. The problem amounts to finding the solution of

$$\begin{array}{c}\underset{{\varvec{Z}}\in A}{\mathrm{max}}-{\sum }_{n=1}^{N}{\sum }_{k=1}^{K}{z}_{kn}{\Vert {{\varvec{x}}}_{n}-{{\varvec{y}}}_{k}\Vert }^{2},\\ A=\left  \{{\varvec{Z}}\left|{\sum }_{k=1}^{K}{z}_{kn}=1\right. \left(n=1,\dots ,N\right), {\sum }_{n=1}^{N}{z}_{kn}\le 1 \left(k=1,\dots ,K\right) \bigg \}.\right.\end{array}$$

This is regarded as a transportation problem where the sum of the squared Euclidean distance between an element feature ${{\varvec{x}}}_{n}$ and a node ${{\varvec{y}}}_{k}$ embedded in the feature space is the cost of transporting one item from source $k$ to destination $n$ under the constraint $A$. We use the lpSolve package^18^ in R^19^ to solve the transportation problem.

This partially modified MCMC is iterated few times (e.g. $T=10$) to make a fine-tuning of the currently given parameters. The assignment variables and the other parameters that exhibit the highest likelihood are chosen to form the final estimate of the PTG. A summary of the algorithm of PTG is shown in Supplementary Algorithm 1.

### Interpretation

The PTG autonomously creates a tabular display of the chemical elements according to the estimated ${\varvec{Z}}$. To understand how the element features such as melting point and electronegativity are compressed on the low-dimensional tabular display, each of the features is mapped onto the resulting table. Specifically, we overlay a smoothed heatmap of each feature on the table. With this PTG property landscape^20^, we can visually understand the distribution of the topographical mapping that indicates how the element features are embedded in the latent space.

### Periodic table as an element descriptor

We consider an evaluation basis for the quality of a designed periodic table in terms of a novel view from data science. A periodic table, including Mendeleev’s classic table, can be considered as one of the most primitive descriptors that encodes known element features into the coordinate system of a low-dimensional latent space. Neighbouring elements on a table should behave similarly and possess similar physicochemical properties. Inspired by such an idea, we consider the use of a periodic table as a descriptor of chemical elements in a task of predicting materials properties based on machine learning^21^. The periodic table is then evaluated quantitatively based on the predictive performance of the descriptor.

For a given table, its coordinates ${{\varvec{u}}}_{k(1)}, \dots , {{\varvec{u}}}_{k(N)}$ of the nodes to which the $N$ elements are assigned are used as a set of element descriptors. For a compound $S$, its fraction of the $N$ elements is denoted by ${w}_{1}\left(S\right),\dots , {w}_{N}\left(S\right)$ where $0\le {w}_{n}\left(S\right)\le 1$ and ${\sum }_{n=1}^{N}{w}_{n}\left(S\right)=1$. The compositional descriptor of $S$ is calculated by ${\varvec{\phi}}\left(S\right)={\sum }_{n=1}^{N}{w}_{n}\left(S\right){{\varvec{u}}}_{k(n)}$. With this descriptor, we derive a prediction model $Y=f\left({\varvec{\phi}}\left(S\right)\right)$, which is trained in $m$ training instances ${\left\{{Y}_{i}, {S}_{i}\right\}}_{i=1}^{m}$, that describes a physicochemical property $Y$ as a function of the descriptor ${\varvec{\phi}}\left(S\right)$ for any given compound $S$. Descriptors exhibiting higher predictability should be recognised as providing more efficient compression performances on the $N$ elements. For comparison, the same analysis was performed using two-dimensional coordinates of the standard periodic table, PCA, and t-SNE, respectively.

### Data: element features

The element feature set was extracted from XenonPy^22^, which is a Python library for materials informatics, by using an Application Programming Interface (API) (see the XenonPy website^23^). The original dataset consisted of 74 features of 118 elements. Since elements with large atomic numbers contained many missing values, we selected 54 elements with the atomic number 1–54 corresponding to hydrogen to xenon that are considered sufficient to retain the periodic rule. After removing features that contained one or more missing values, the dataset was reduced to 39 features of 54 elements. For the 54 × 39 data matrix, each feature (column) was standardized to have mean 0 and variance 1. A heatmap display of the data matrix and a detailed description of the 39 features are provided in Supplementary Fig. S1 and S2, respectively.

### Analysis procedure

We performed the PTG on two different layouts of nodes, square, and three-dimensional conical layouts. In the square layout of $L=2$, we set $K=25$ in the first step of PTG in which the $5\times 5$ nodes were evenly arranged on the area $\left[-1, 1\right]\times \left[-1, 1\right]$. In the second step, we increased the number of nodes to $9\times 9$ by placing new nodes at the middle points of the line segments connecting between each node. In the conical layout of $L=3,$ we first used a set of nodes with $K=25$ that were arranged uniformly on the surface of the cone placed in the area $\left[-1, 1\right]\times \left[-1, 1\right]\times \left[-1, 1\right]$. The cone was sliced into 4 sections in the same height along the vertical axis. Then, 1 (vertex), 4, 8, and 12 (bottom) nodes were uniformly placed on the outer part of the 4 cut surfaces. In the next step, the number of slices was increased by 7, and 1 (vertex), 4, 8, 12, 16, 20, and 24 (bottom) nodes were uniformly arranged in the same way. In both the cases, we set ${{\varvec{\xi}}}_{g}={{\varvec{\xi}}}_{r}=\left(1/3, 3\right)$, the number of iteration in MCMC was set to $T=\mathrm{10,000}$ with the burn-in step ${T}_{b}=5000$, and the number of iteration in the third step of fine-tuning was set to $T=10$. See the Supplementary Information section for further details on the hyperparameter settings and analysis procedure.

The PTG algorithm was implemented using R codes, which are available at^24^ with the element dataset. Readers can run the PTG algorithm with the element data used in this paper. As a demonstration, the PTG was performed on another three different layouts: a rectangular table with $5\times 18$ equally spaced grids, which is same to the layout of the standard periodic table, and two three-dimensional layouts taking the forms of cylinder, and cubic, respectively. The results are shown in Fig. S8.

## Results

### Results of PTG

#### Square table

**Figure 2:**
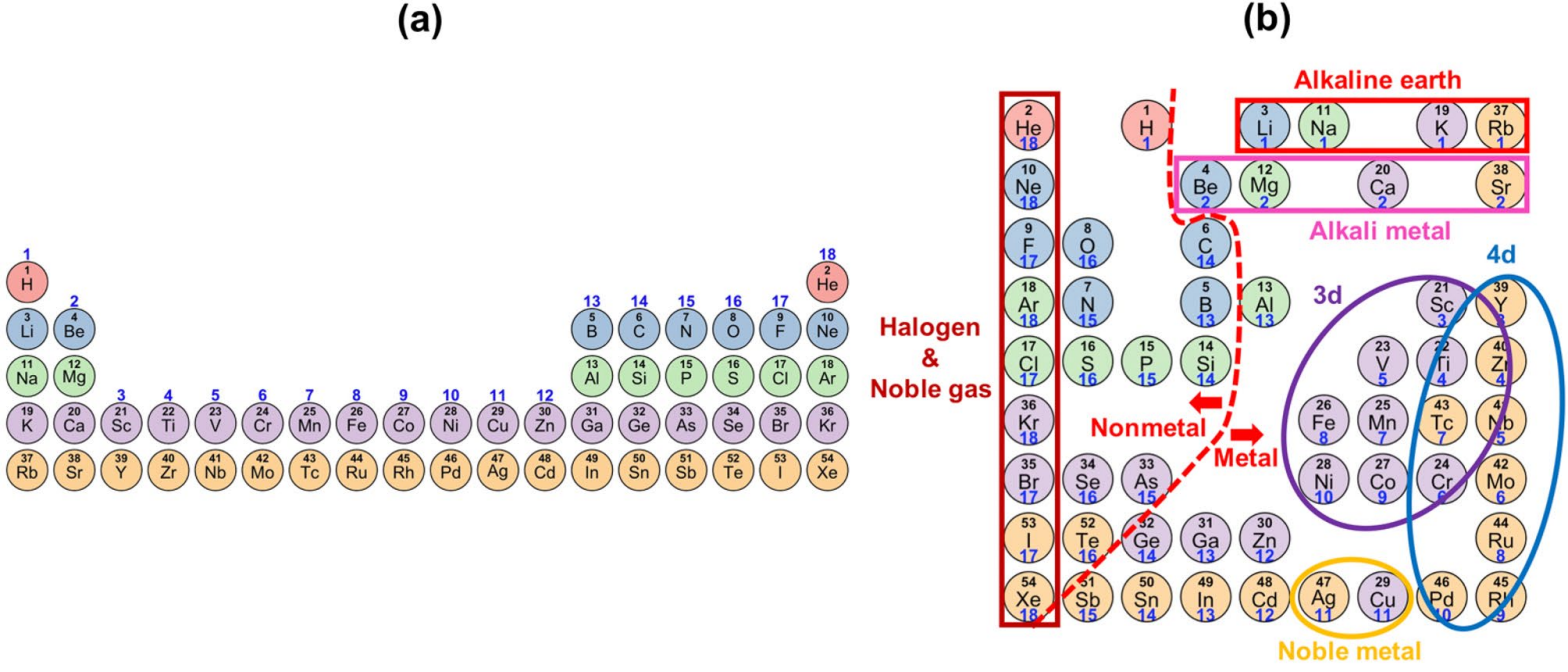
(**a**) The currently most common periodic table of the elements. (**b**) Square PTG table created from the training data of 39 features of the 54 elements. The elements are colour-coded by periods and numbered by atomic numbers. The number shown in blue below each element symbol represents the group number (the column in the standard periodic table).

Figure 2 shows a PTG-created layout of the 54 elements on the $9\times 9$ square lattice. Elements in each period of the standard periodic table were configured in a fan shape from the top left to the bottom right. The elements in the square table are clearly separated into metal and nonmetal by the red dashed line shown in Fig. 2. The 3d and 4d transition elements were separated and both clustered in the lower right. In addition, the elements were clearly clustered by groups such as alkali metals, alkaline earth metals, halogens, and noble gases. This looked like a variant of the original periodic table: the original table was folded around the centre on which transition elements are positioned, the two separated blocks of group 1–2 and 13–18 in the first to third periods were brought nearer with each other while keeping away from the area of transition elements, and they were stored into the square table. Notably, the square table exhibited the discontinuity from group 18 to group 1 as in the original table. Though results are not shown, the same discontinuity appeared frequently in most square tables created in the experiments under different conditions.

#### Conical table

**Figure 3:**
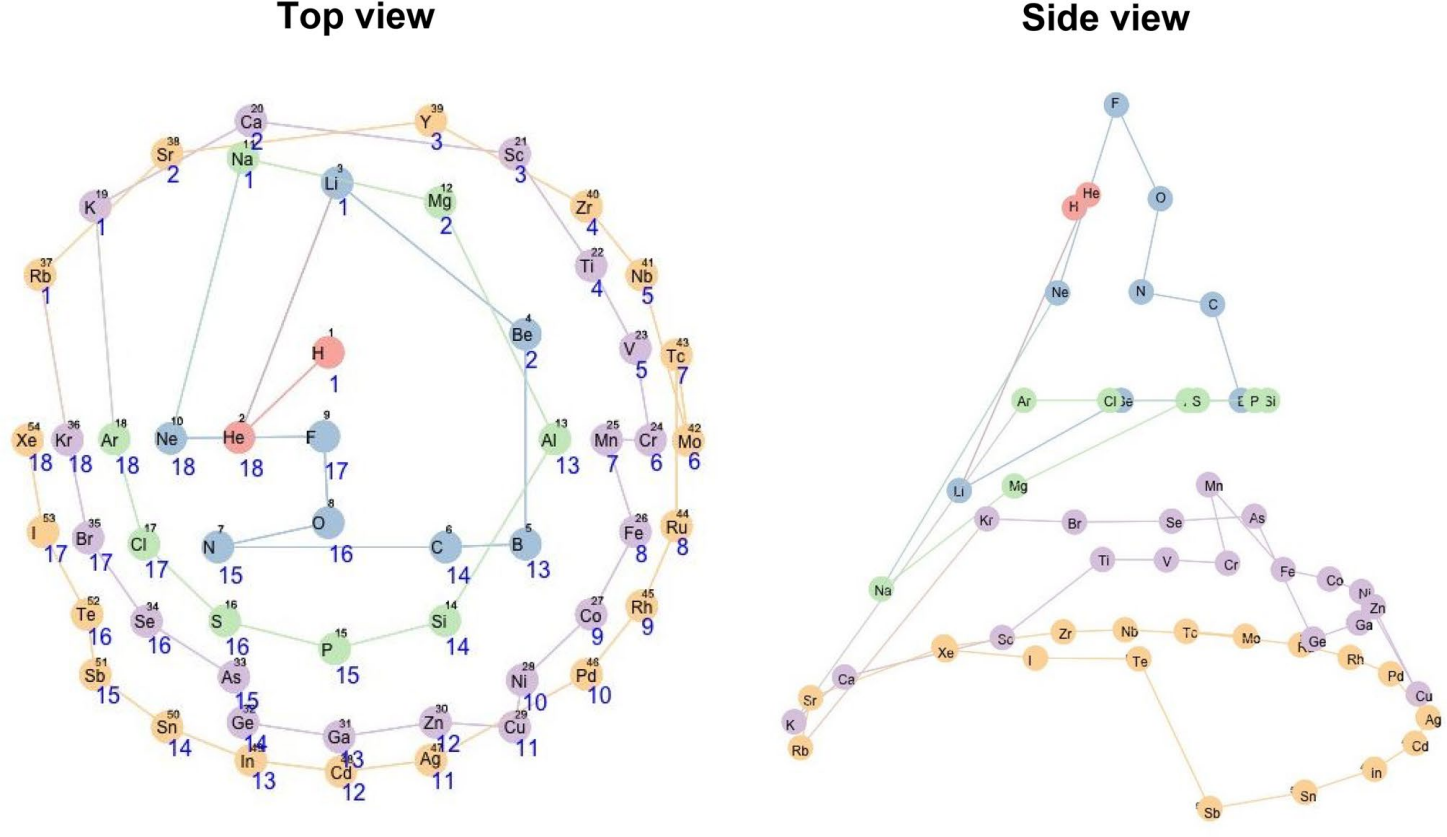
Created conical table of 54 chemical elements. The elements are colour-coded according to five periods and numbered by atomic numbers. A line passing through the elements is drawn in the order of atomic numbers. The number shown in blue below each element symbol represents the group number (the column in the standard periodic table). The left and right figures show the same table viewed from top and side, respectively.

**Figure 4:**
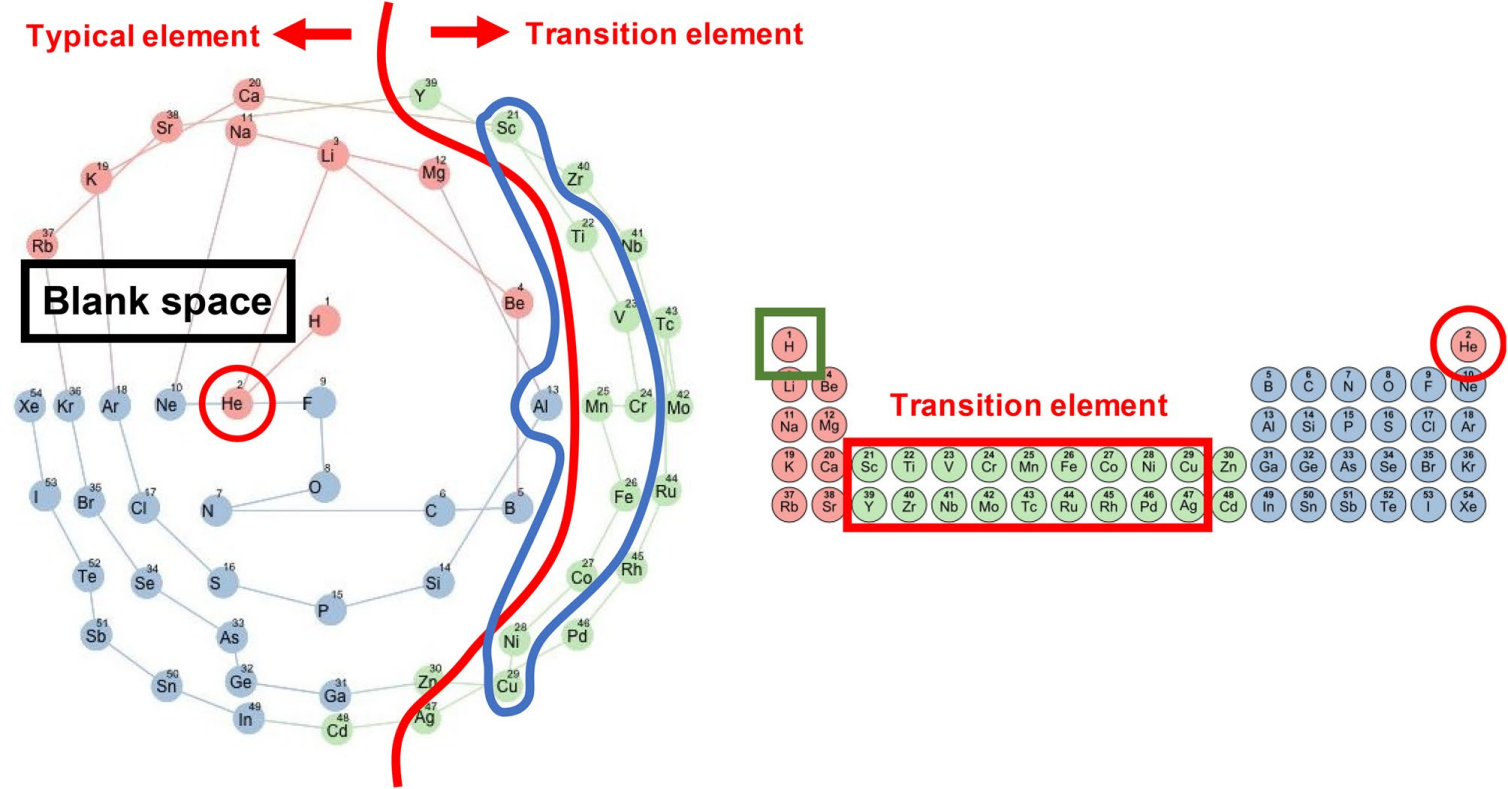
The left panel shows a conical table viewed from above. The elements are colour-coded according to three blocks in the standard periodic table that are indicated in the right panel. The red line in the left indicates the segment between transition elements and typical elements.

Figure 3 shows a layout on the three-dimensional conical nodes. The elements were arranged in a spiral structure starting from the top of the cone according to increasing atomic numbers. Viewed from the top, the elements were stratified concentrically by the periods of the standard periodic table. This view was slightly similar to the circular periodic table that was constructed in a different study^7^. One block corresponded to a set of elements divided according to the orbital type of the electrons of the highest energy levels. In the standard periodic table, helium (He: an element circled by the red line in Fig. 4) is located away from the other s-block elements (a set of elements coloured red in Fig. 4), but in the conical table, it was located close to them. It was also seen that the elements in the conical table were clearly classified into typical elements and transition elements by the red line shown in Fig. 4. A blank space was observed between group 1 and group 18 on the conical table implying that there is a gap of properties between them in the feature space.

In the spiral structure viewed from above, the atomic numbers were monotonically arranged from top to bottom except for a few elements. The disorder appeared in group 6 to 7: chromium (Cr: atomic number = 24) and manganese (Mn: 25) in period 4 or molybdenum (Mo: 42) and technetium (Tc: 43) in period 5. In the conical table, the elements were arranged radially according to groups, and elements of group 1 and 2 were located a little away from group 3.

### Interpretation

**Figure 5:**
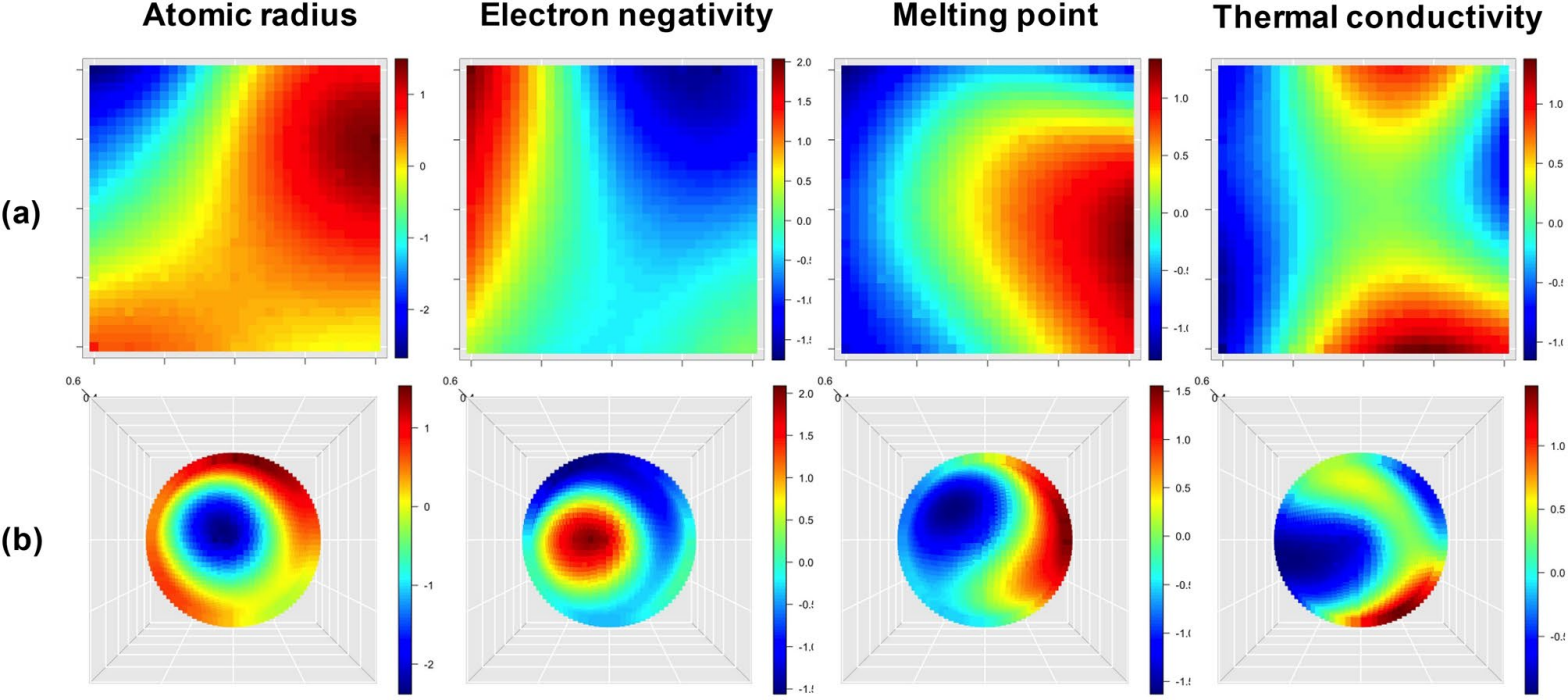
Property landscapes of atomic radius (Rahm et al.^27^), electron negativity, melting point, and thermal conductivity at 25 ℃ that are embedded in the latent spaces. The heatmaps are laid on (**a**) the square table in Fig. 2 and (**b**) the conical table (top view) in Fig. 3.

To understand how the element features have been embedded on the created tables, each of the features was mapped on the lower-dimensional latent space (Fig. 5). In the property landscape of the conical table, atomic radius increased gradually and concentrically from the top of the cone, electron negativity decreased gradually and concentrically from the top of the cone, and melting point gradually increased from right to left. The distribution of thermal conductivity looked a little more complicated than the former three, but continuity and unimodality still held on the surface of the three-dimensional conical table. On the other hand, in the square table, the landscapes of some element features, e.g. atomic radius and thermal conductivity, exhibited multimodality. This discontinuity arose from the unnatural layout of the elements in the two-dimensional tabular representation as in the standard periodic table. The PTG property landscapes of the 39 features are shown in the Supplementary Information section.

### Quantitative comparison of periodic tables

To evaluate the validity of a periodic table and uncover the information gain and loss of the reduced representation, we considered the use of a table as an element descriptor in machine learning tasks. The task to be addressed was the prediction of formation energies of inorganic compounds. The dataset that we used for the training of random forest regressors (RF)^25^ was obtained from Materials Project^26^, which is a database of materials properties generated from high-throughput first-principles calculations. Among all inorganic compounds in Materials Project, we selected compounds that are stable and consist of elements with the atomic number 1–54 (H to Xe). The dataset consisted of the formation energies per atom of 12,373 inorganic compounds.

The objective here was to train an RF that describes the formation energy as a function of the conical descriptor ${\varvec{\phi}}\left(S\right)$ obtained by composing $S$ and the three-dimensional coordinates of the elements in the conical table. This is described in the Methods section above. For comparison, we built four different models using the descriptors based on the two-dimensional coordinates in the created square table, the standard periodic table, PCA, and t-SNE, respectively.

**Figure 6:**
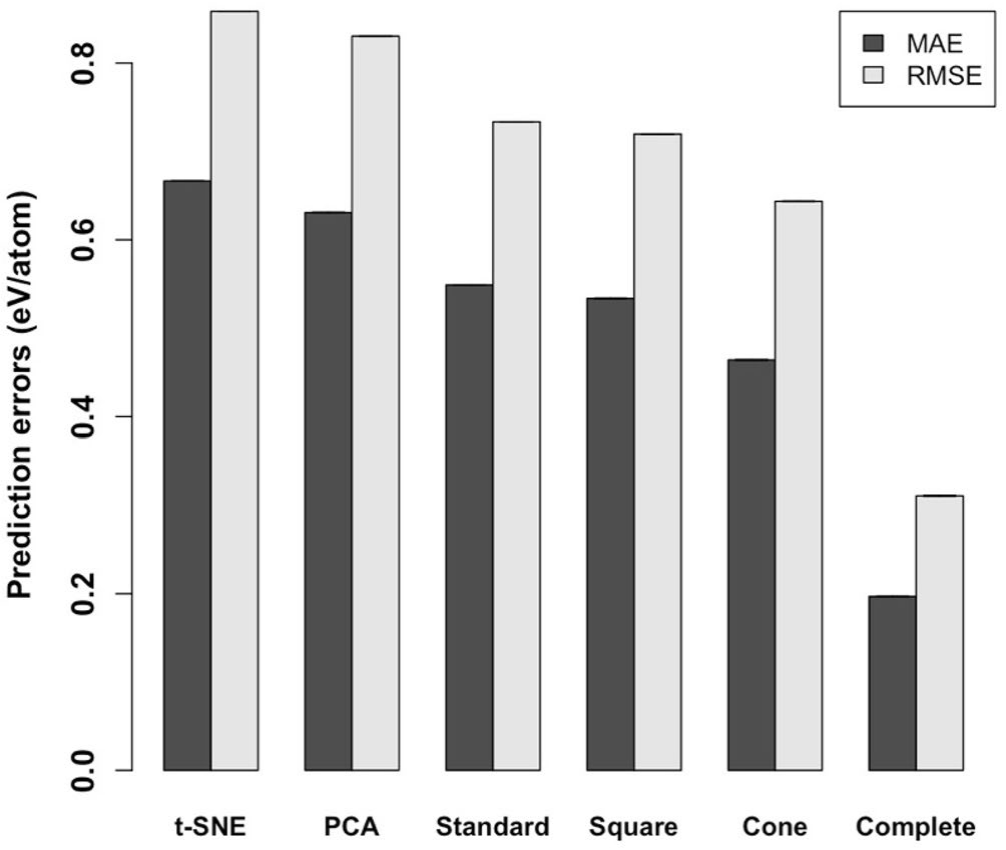
Performance of the prediction of the formation energy per atom for the models using six different descriptors. The vertical axis indicates cross-validated MAE and RMSE of RF regressors trained with the six different descriptors obtained from the coordinates of elements in the representation made by t-SNE and PCA (corresponding to top-left and top-right in Fig. S3, respectively), the standard periodic table, the square PTG table, the conical PTG table, and the complete set of the 39-dimensional feature that were used to build the PTG table, respectively. The error bars denote the standard deviations in five independent trials of the cross-validation (the error bars are invisible because of substantially small scales).

We performed the five-fold cross-validation on the 12,373 samples for the six types of descriptors. As shown in Fig. 6, the conical PTG achieved a mean absolute error (MAE) of 0.464 eV/atom and a root mean square error (RMSE) of 0.643 eV/atom, whereas the MAE and the RMSE of the square PTG and the standard periodic table were 0.533 eV/atom and 0.719 eV/atom, and 0.549 eV/atom and 0.734 eV/atom, respectively. The models based on PCA and t-SNE gave the MAE of 0.631 eV/atom and 0.667 eV/atom respectively, and the RMSE of 0.830 eV/atom and 0.859 eV/atom respectively, which were clearly less accurate in their predictions. Finally, the model based on the complete set of the 39-dimensional feature gave the MAE of 0.197 eV/atom and the RMSE of 0.311 eV/atom (This is added to show how the overall information being retained by the tables). In summary, the square PTG was slightly superior to the standard periodic table, but the conical PTG table outperformed the standard periodic table, the square PTG, PCA, and t-SNE, respectively.

**Figure 7:**
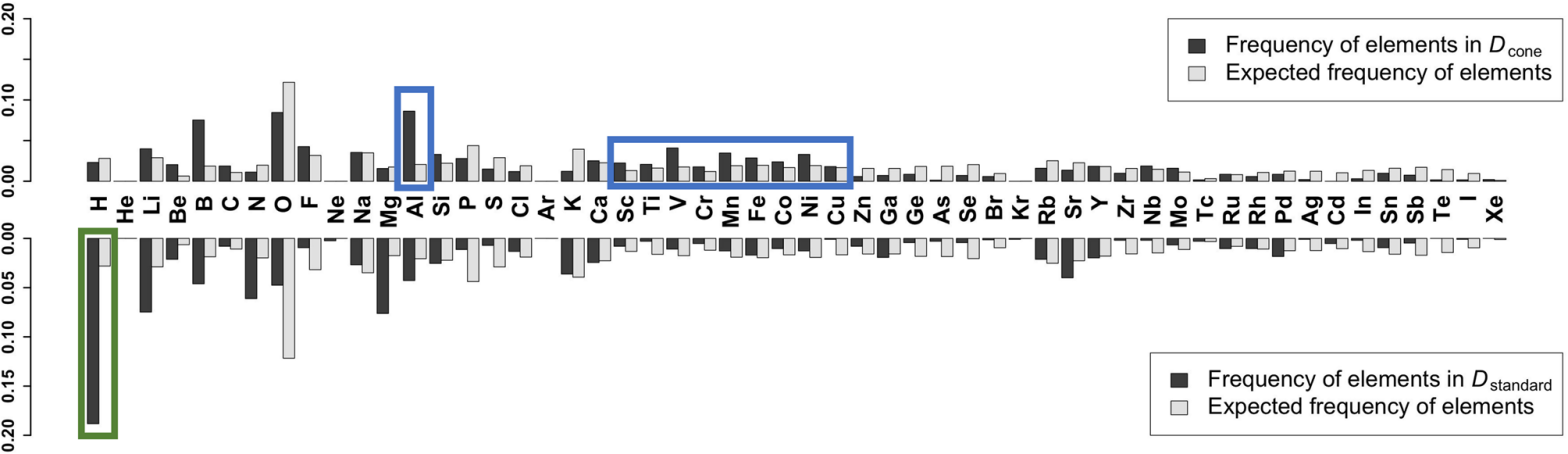
Comparison of the frequencies of chemical elements in ${D}_{\mathrm{cone}}$ (top: black bar chart) and ${D}_{\mathrm{standard}}$ (bottom: black bar chart). White bar charts show the expected frequency calculated with the number of occurrences in the overall population.

A detailed investigation of the prediction results provided some insights into the difference in information compression between the three-dimensional conical table and the standard periodic table. We focused on a subset of the compounds used in the validation, hereafter denoted by ${D}_{\mathrm{cone}}$ (i.e. the conical descriptor dominant set), that had the MAE values less than 0.3 eV/atom for the conical descriptor, but 1.0 eV/atom greater than the conical descriptor for the standard periodic table. Likewise, we identified ${D}_{\mathrm{standard}}$ with the MAE values less than 0.3 eV/atom for the standard periodic table, but 1.0 eV/atom greater than the standard periodic table for the conical table. We counted the frequency of a chemical element in ${D}_{\mathrm{cone}}$ and ${D}_{\mathrm{standard}}$, and evaluated the enrichment of the element by comparing its expected frequency calculated with the background, i.e. the number of occurrence in the overall population (the 12,373 compounds in Materials Project). As shown in Fig. 7, a significantly enriched group in ${D}_{\mathrm{cone}}$ comprised transition elements in the fourth period that correspond to atomic number 21–29. Aluminium (Al) was also enriched in ${D}_{\mathrm{cone}}$ (Fig. 7: a set of elements circled by a blue line). Notably, these over-represented elements formed a cluster in the created conical table (Fig. 4: a set of elements circled by a blue line). On the other hand, hydrogen (H) was significantly enriched in ${D}_{\mathrm{standard}}$ (Fig. 7: an element circled by green line). H is located just above lithium (Li) in the standard periodic table (Fig. 4: an element circled by a green line), while it was located between fluorine (F) and Li in the conical periodic table.

## Concluding remarks

Since the emergence of Mendeleev’s periodic table, hundreds of redesigned tables have been created. In terms of machine learning, the tabular construction can be considered a task of reducing the dimensionality of high-dimensional data. A previous study first attempted to yield the periodic table using machine learning by applying SOM to five element features available in Mendeleev’s time^9^. Though the SOM successfully placed similarly behaved elements in neighbouring sub-regions on the table, the reported results still never reached Mendeleev’s achievement as it obviously failed to capture the underlying periodicity of the elements. To reach Mendeleev’s achievement, we attempted to develop PTG as an unsupervised machine learning algorithm that can automate the translation of high-dimensional data into a tabular form with varying layouts on-demand. The proposed method is applicable as long as a feature set and a template of the table are given. The task of compiling data into tabular displays is the most basic task in data analysis. Nonetheless, there has been much less research of this kind so far in data science.

In the previous study based on SOM, some chemical elements having similar properties occupied the same cell in the table due to SOM inability to guarantee non-overlapping assignments of elements. When we began this study, there had been no existing machine learning methods for the task of tabular construction. To the best of our knowledge, the PTG algorithm that we present is the first tabular constructor based on machine learning, yet this is a secondary contribution of this study.

In this study, we created the two types of periodic tables with three additional layouts. The square table was considerably similar to the currently most common periodic table, but some outstanding differences were observed, for example in the arrangement of H and He. These elements were placed far away in the standard periodic table, but their physicochemical properties were similar. The PTG suggested that these elements should be put closer according to the observed data. The three-dimensional layout on the cone also provided some insight into how the transition elements in the fourth period, including aluminium (Al), should be arranged. In addition, the created conical table provided a re-ordering from Cr to Mn in period 4 and from Mo to Tc in period 5 in the standard table.

A periodic table is the most basic descriptor of chemical elements. Historically, the primary design objective has focused on the understandability and the interpretability to humans even at the expense of reducing some key detailed features. Here, we provided a new way of looking at periodic tables. The coordinates of elements put on a table can be considered as an element descriptor, which is also converted to a descriptor of materials. The quality of designed tables should be assessed on the performance of predicting physicochemical properties of resulting machine learning models. This study focused only on the prediction of formation energies, but more diverse properties should be incorporated into the design objective. Also, we focused only on the two types of layouts, but there are a lot of options for potentially promising layouts. Our algorithm would contribute to the recreation of more sophisticated tabular displays of chemical elements.

## Supplementary Information

Supplementary Information.

## References

[1] Mendeleev D (1869). On the relationship of the properties of the elements to their atomic weights. Zeitschrift für Chemie.

[2] Moseley HGJ (1913). The high frequency spectra of the elements. Philos. Mag..

[3] Bohr N (1913). On the constitution of atoms and molecules. Philos. Mag..

[4] Marchese, F. T. The chemical table: an open dialog between visualization and design. In *12th International Conference Information Visualisation*. 75–81. 10.1109/IV.2008.79 (2008).

[5] The internet database of periodic tables https://www.meta-synthesis.com/webbook/35_pt/pt_database.php.

[6] Scerri E (2012). Trouble in the periodic table. Educat. Chem..

[7] Abubakr, M. An alternate graphical representation of periodic table of chemical elements. https://arxiv.org/pdf/0910.0273.pdf (2009).

[8] Katz G (2001). The periodic table: an eight period table for the 21st century. Chem. Educat..

[9] Lemes MR, Pino AD (2011). Periodic table of the elements in the perspective of artificial neural networks. J. Chem. Educat..

[10] Kohonen T (1982). Self-organized formation of topologically correct feature maps. Biol. Cybern..

[11] Schölkopf, B., Smola, A. & Müller, K. R. Kernel principal component analysis. In *International Conference on Artificial Neural Networks* 583–588 (Springer, 1997).

[12] Tenenbaum JB, Silva V, Langford JC (2000). A global geometric framework for nonlinear dimensionality reduction. Science.

[13] Roweis ST, Saul LK (2000). Nonlinear dimensionality reduction by locally linear embedding. Science.

[14] Maaten L, Hinton G (2008). Visualizing data using t-sne. J. Mach. Learn. Res..

[15] Bishop CM, Svensén M, Williams CKIGTM (1998). The generative topographic mapping. Neural Comput..

[16] Yamaguchi, N. GTM with latent variable dependent length-scale and variance. In *International Automatic Control Conference (CACS)* 532–538 (IEEE, 2013).

[17] Williams CKI, Jordan MI (1997). Prediction with Gaussian process: from linear regression to linear prediction and beyond. Learning in Graphical Models. NATO ASI Series (Series D: Behavioural and Social Sciences).

[18] Berkelaar, M. & Others. R package ‘lpSolve’. CRAN (2015).

[19] R Development Core Team. R: a language and environment for statistical computing. http://www.R-project.org (2013).

[20] Gaspar HA, Baskin CE, Marcou G (2015). Chemical data visualization and analysis with incremental generative topographic mapping: big data challenge. J. Chem. Inf. Model..

[21] Zhou Q (2018). Learning atoms for materials discovery. Proc. Natl. Acad. Sci. USA.

[22] Xenonpy http://xenonpy.readthedocs.io/en/latest/ (2019).

[23] https://github.com/yoshida-lab/XenonPy/blob/master/samples/dataset_and_preset.ipynb.

[24] https://github.com/Minoru938/PTG.

[25] Breiman L (2001). Random forests. Mach. Learn..

[26] Materials Project https://materialsproject.org/ (2019).

[27] Rahm M, Hoffmann R, Ashcroft NW (2016). Atomic and ionic radii of elements. Chem. Eur. J..

