# Supplementary material for: Recreation of the periodic table with an unsupervised machine learning algorithm
Source: Sci Rep. 2021 Feb 26;11:4780. doi: 10.1038/s41598-021-81850-z (PMC7910619; doi:10.1038/s41598-021-81850-z)
Supplement: Supplementary file 1 — Supplementary Information. [file 41598_2021_81850_MOESM1_ESM.pdf]

Supplementary Information

Recreation of the Periodic Table with an Unsupervised Machine Learning Algorithm

Minoru Kusaba[1,*], **Chang Liu**[2], **Yukinori Koyama**[3], **Kiyoyuki Terakura**[4], **Ryo Yoshida**[1,2,3,*]

[1]The Graduate University for Advanced Studies, SOKENDAI, Tachikawa, Tokyo 190-8562, Japan
[2]The Institute of Statistical Mathematics, Research Organization of Information and Systems, Tachikawa, Tokyo 190-8562, Japan
[3]National Institute for Materials Science, Tsukuba, Ibaraki 305-0047, Japan
[4]National Institute of Advanced Industrial Science and Technology, Tsukuba, Ibaraki 305-8560, Japan
[*]kusaba@ism.ac.jp, yoshidar@ism.ac.jp.

Fig. S1 shows a heatmap of the elements' data used in this study. A detailed description of the elements-level properties is given in Fig. S2. Fig. S3 shows visualization results of the elements' data on the two-dimensional space using various unsupervised learning methods. The periodic table generator (PTG) landscapes of 39 features for the square and conical tables corresponding to Fig. 2 and 3 are shown in Fig. S4 and S5. From pages 9 to 12, a detailed description of GTM-LDLV is given. A summary of the algorithm of PTG is shown in Algorithm 1. Details of the analysis procedure in this study are given from pages 14 to 16. On page 16 to 18, we show results of the PTG with another three layouts of nodes: rectangle, three-dimensional cylinder, and cubic layouts, respectively.

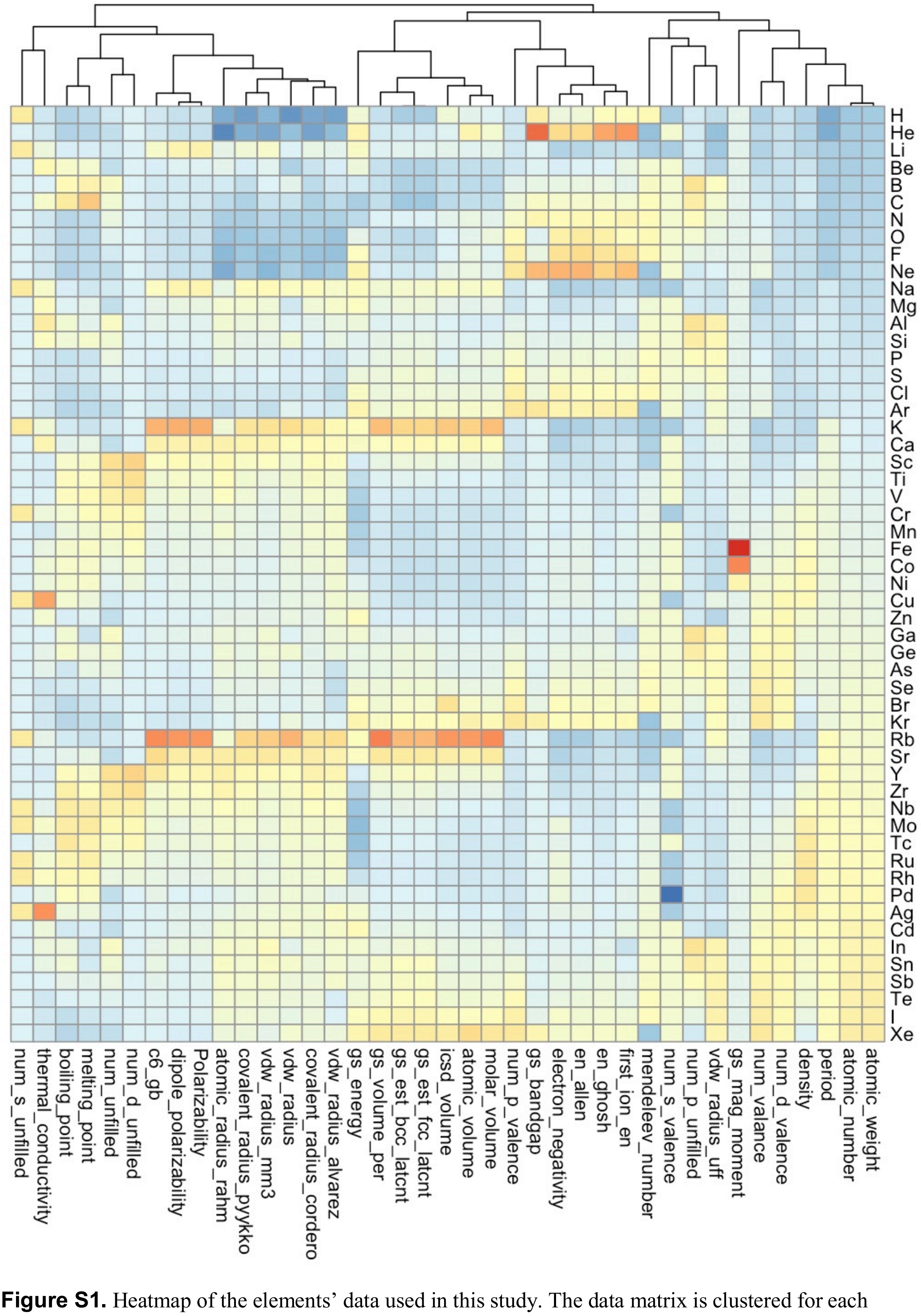

Figure S1. Heatmap of the elements' data used in this study. The data matrix is clustered for each column (features).

Elements-level properties used for analysis

feature	description
atomic_number	Number of protons found in the nucleus of an atom
atomic_radius_rahm	Atomic radius by Rahm et al
atomic_volume	Atomic volume
atomic_weight	The mass of an atom
boiling_point	Boiling temperature
c6_gb	C_6 dispersion coefficient in a.u
covalent_radius_cordero	Covalent radius by Cerdero et al
covalent_radius_pyykko	Single bond covalent radius by Pyykko et al
density	Density at 295K
dipole_polarizability	Dipole polarizability
electron_negativity	Tendency of an atom to attract a shared pair of electrons
en_allen	Allen’s scale of electronegativity
en_ghosh	Ghosh’s scale of electronegativity
first_ion_en	First ionisation energy
gs_bandgap	DFT bandgap energy of T=0K ground state
gs_energy	DFT energy per atom (raw VASP value) of T=0K ground state
gs_est_bcc_latcnt	Estimated BCC lattice parameter based on the DFT volume
gs_est_fcc_latcnt	Estimated FCC lattice parameter based on the DFT volume
gs_mag_moment	DFT magnetic momenet of T=0K ground state
gs_volume_per	DFT volume per atom of T=0K ground state
icsd_volume	Atom volume in ICSD database
mendeleev_number	Atom number in mendeleev’s periodic table
melting_point	Melting point
molar_volume	Molar volume
num_unfilled	Total unfilled electron
num_valence	Total valence electron
num_d_unfilled	Unfilled electron in d shell
num_d_valence	Valence electron in d shell
num_p_unfilled	Unfilled electron in p shell
num_p_valence	Valence electron in p shell
num_s_unfilled	Unfilled electron in s shell
num_s_valence	Valence electron in s shell
period	Period in the periodic table
thermal_conductivity	Thermal conductivity at 25 C
vdw_radius	Van der Waals radius
vdw_radius_alvarez	Van der Waals radius according to Alvarez
vdw_radius_mm3	Van der Waals radius from the MM3 FF
vdw_radius_uff	Van der Waals radius from the UFF
Polarizability	Ability to form instantaneous dipoles

Figure S2. Detailed description for 39 elements-level features used in this analysis.

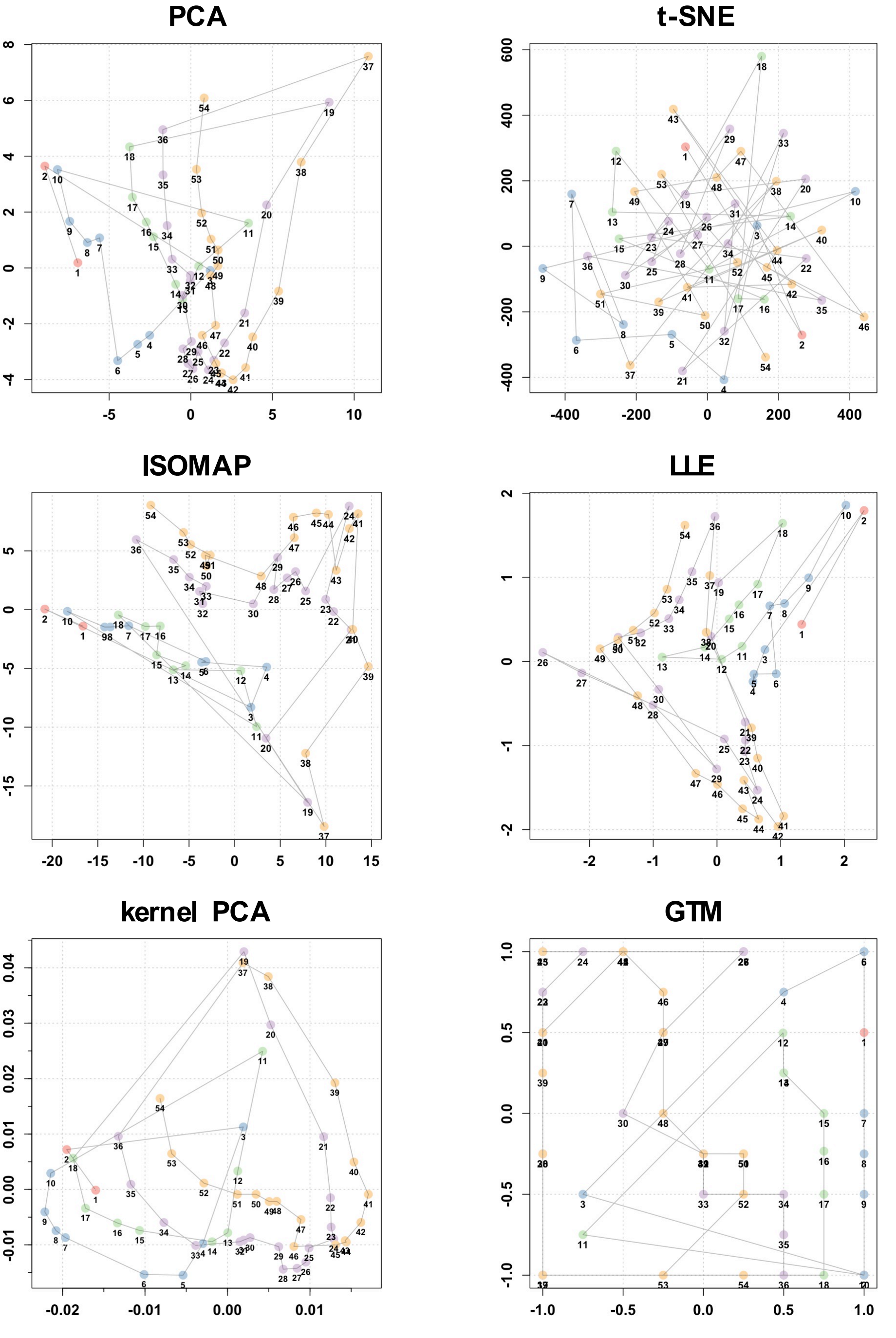

Figure S3. Visualization results of the elements' data on the two-dimensional space using PCA (top-left), t-SNE (top-right), ISOMAP with neighbors = 3 (middle-left), LLE with neighbors = 9 (middle-right), kernel PCA with ANOVA kernel and sigma = 0.2 (bottom-left), and GTM with $K = 9 \times 9$ grid points and 16 basis functions (bottom-right). The elements are colour-coded by periods and numbered by atomic numbers. A line passing through the elements is drawn in the order of atomic numbers.

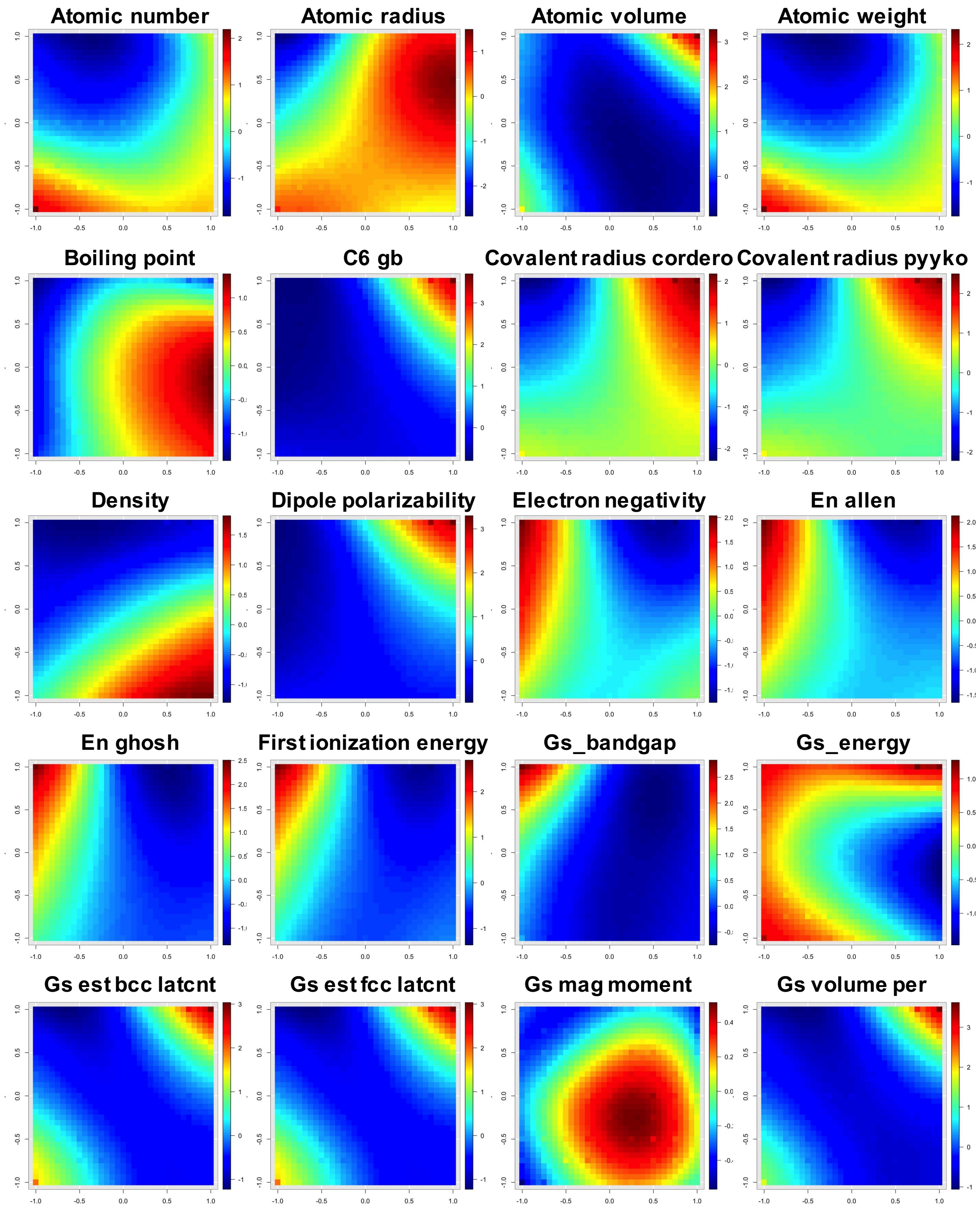
Atomic number
Atomic radius
Atomic volume
Atomic weight
Boiling point
C6 gb
Covalent radius cordero
Covalent radius pyyko
Density
Dipole polarizability
Electron negativity
En allen
En ghosh
First ionization energy
Gs_bandgap
Gs_energy
Gs est bcc latcnt
Gs est fcc latcnt
Gs mag moment
Gs volume per

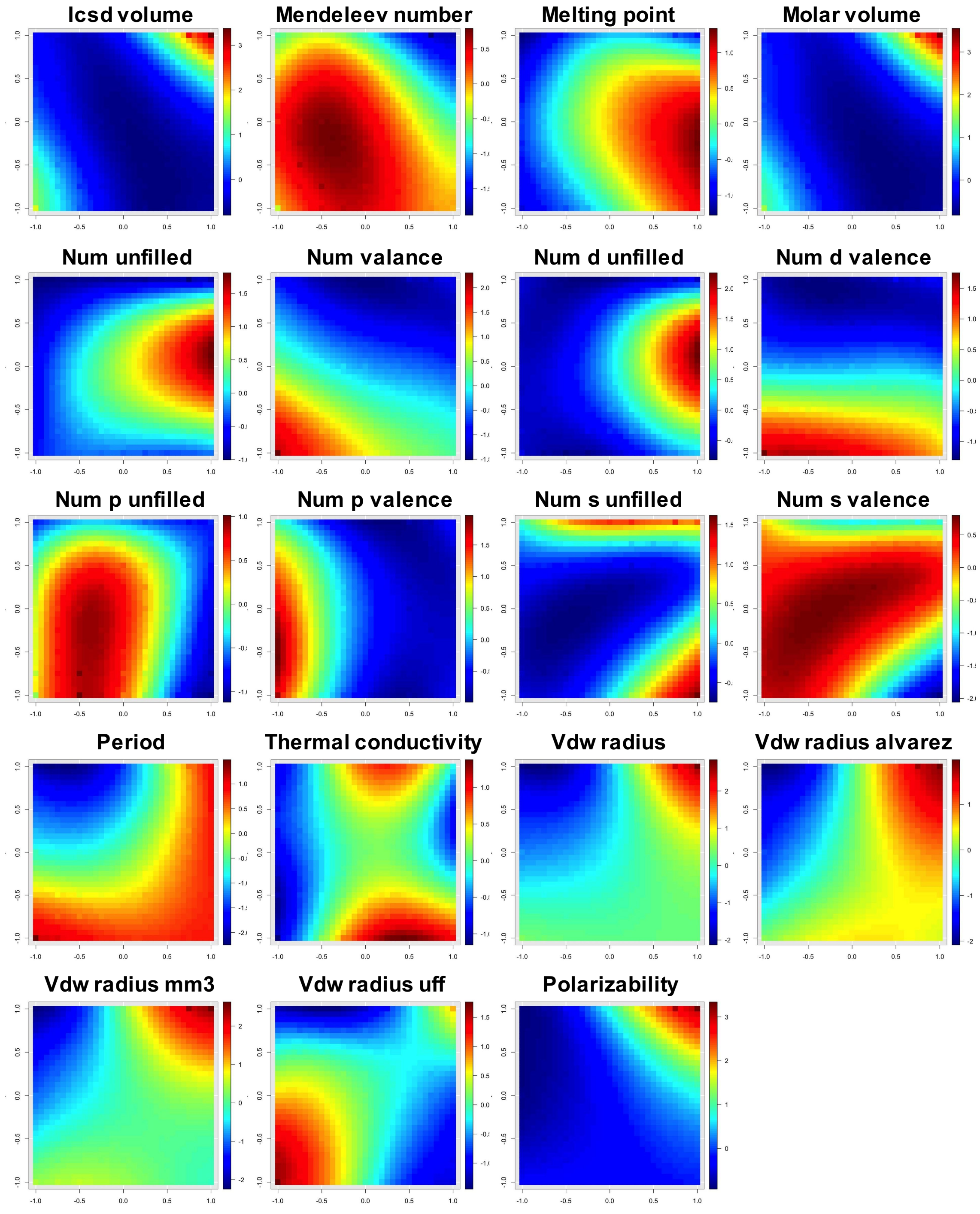

Figure S4. PTG property landscape of all 39 features for the square PTG table shown in Fig. 2.

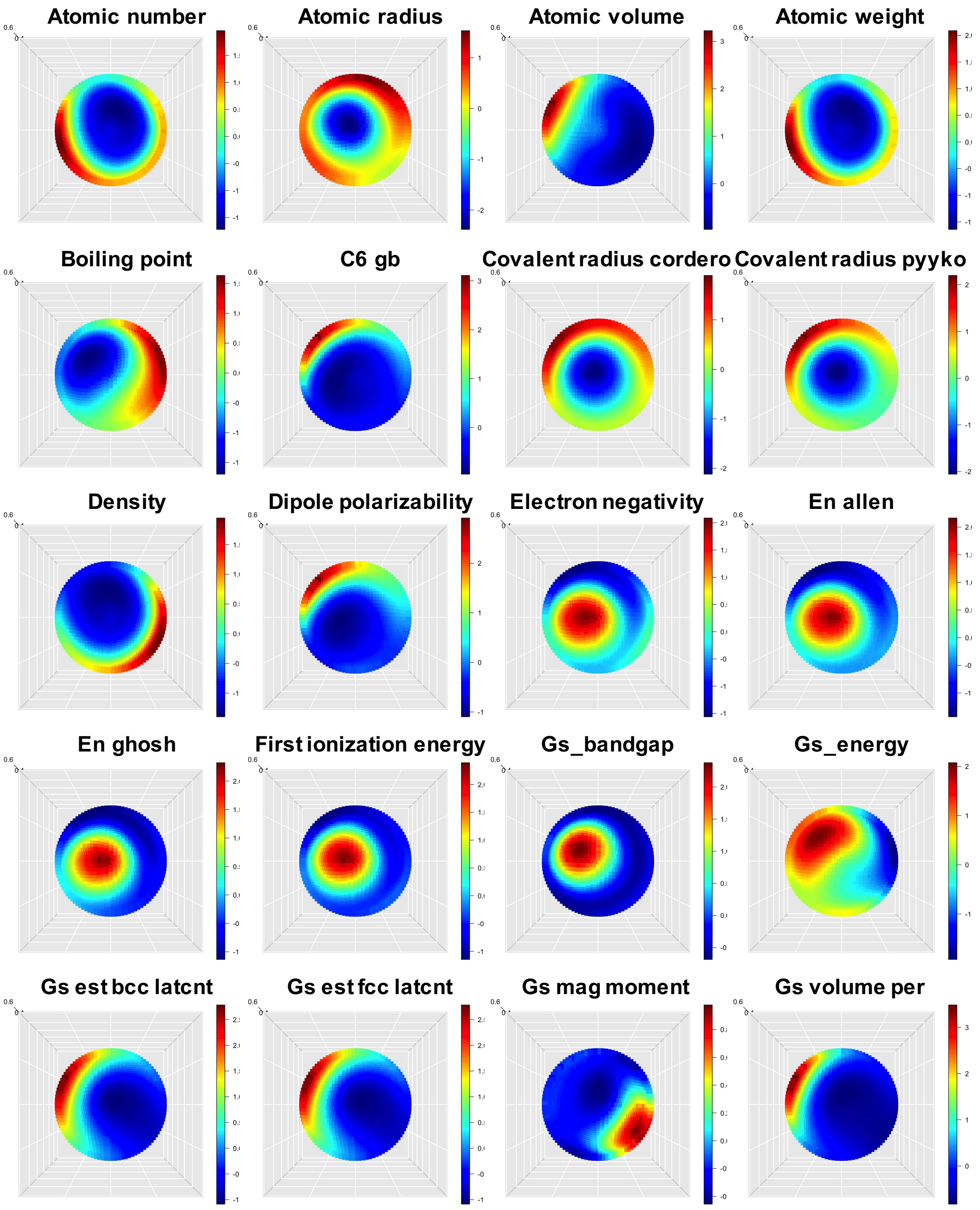
Atomic number
Atomic radius
Atomic volume
Atomic weight
Boiling point
C6 gb
Covalent radius cordero
Covalent radius pyyko
Density
Dipole polarizability
Electron negativity
En allen
En ghosh
First ionization energy
Gs_bandgap
Gs_energy
Gs est bcc latcnt
Gs est fcc latcnt
Gs mag moment
Gs volume per

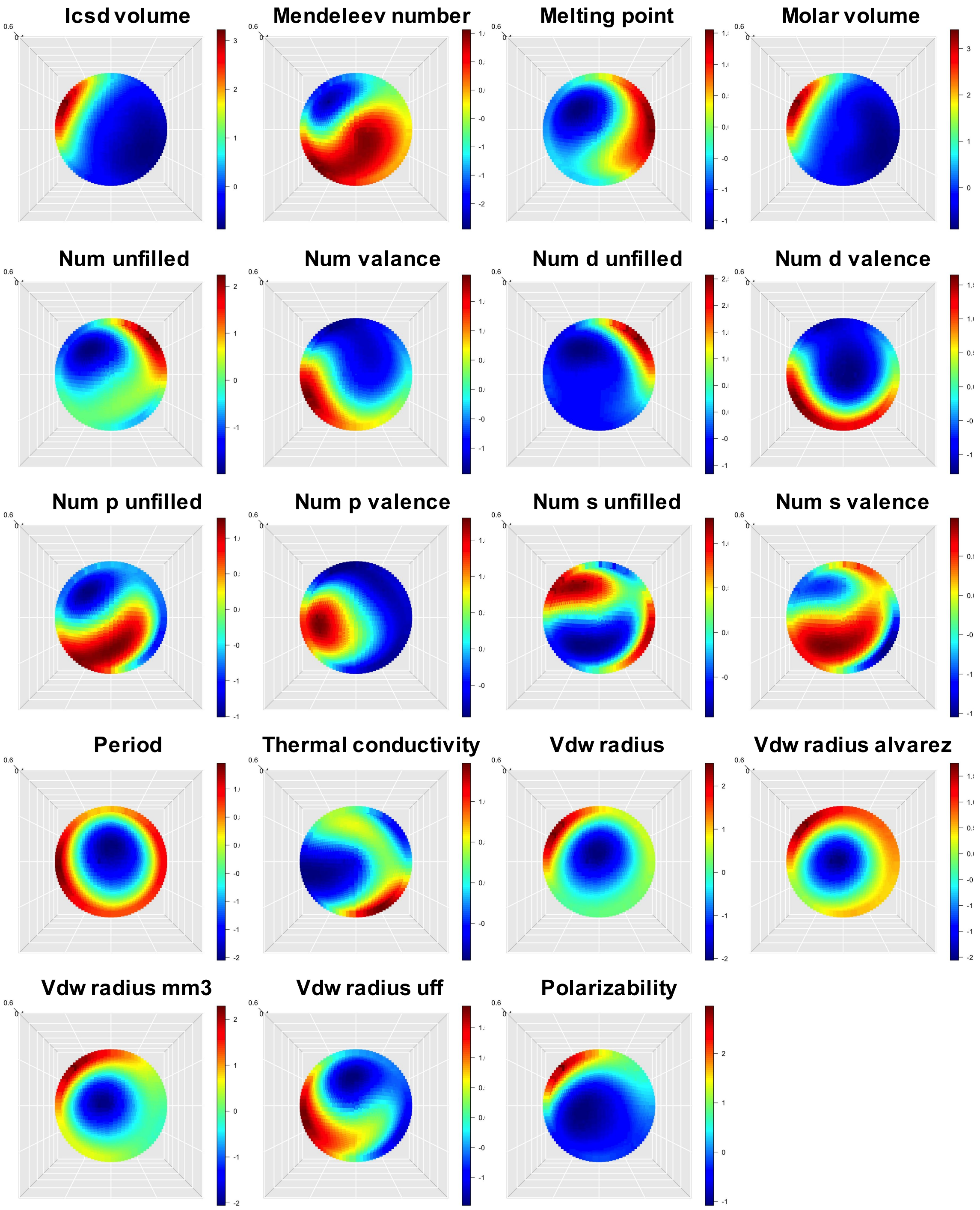

Figure S5. PTG property landscapes of all 39 features for the conical PTG table shown in Fig. 3.

Detailed Method of GTM-LDLV

Our learning method can be considered as an extension of generative topographic mapping (GTM) proposed by Bishop et al. [1]. GTM is a latent variable model that represents the probability density of data using a nonlinear function of lower dimensional latent variables. It can be regarded as a stochastic formulation of self-organizing map (SOM) [2].

In GTM, K grid points (called "nodes" hereafter) $\boldsymbol{u}_1, \cdots, \boldsymbol{u}_K$ regularly arranged in the L-dimensional latent space are prepared for data visualization, and consider a nonlinear function $\boldsymbol{f}(\boldsymbol{u}_k; \boldsymbol{\theta})$ that maps the nodes $\boldsymbol{u}_k$ to a point $\boldsymbol{y}_k$ on the D-dimensional feature space. The dimension of the latent space L is set less than 3 for visualization. $\boldsymbol{\theta}$ is a parameter set that determines $\boldsymbol{f}(\boldsymbol{u}_k; \boldsymbol{\theta})$. It is assumed that the D-dimensional feature vector $\boldsymbol{x}_n$ is generated independently by a restricted mixture of K Gaussian distributions, where all mixing coefficients are $1/K$, the mean of the Gaussian distribution is $\boldsymbol{y}_k$, and the covariance matrix is all $\beta^{-1}\boldsymbol{I}$. Then, the distribution is given by

$$p(\boldsymbol{x}_n | \boldsymbol{\theta}, \beta) = \frac{1}{K} \sum_{k=1}^{K} p(\boldsymbol{x}_n | \boldsymbol{u}_k, \boldsymbol{\theta}, \beta),$$

$$p(\boldsymbol{x}_n | \boldsymbol{u}_k, \boldsymbol{\theta}, \beta) = N(\boldsymbol{x}_n | \boldsymbol{y}_k, \beta^{-1}\boldsymbol{I}), \qquad \boldsymbol{y}_k = \boldsymbol{f}(\boldsymbol{u}_k; \boldsymbol{\theta}),$$

where $N(\cdot | \boldsymbol{\mu}, \boldsymbol{\Sigma})$ denotes the Gaussian density function with mean $\boldsymbol{\mu}$ and covariance matrix $\boldsymbol{\Sigma}$. Here, we introduce a vector of K latent variables, $\boldsymbol{z}_n = (z_{1n}, \cdots, z_{Kn})'$. The kth entry z_{kn} takes the value 1 if $\boldsymbol{x}_n$ is generated by the kth component distribution, and 0 otherwise. Here, let $\boldsymbol{X}$ denote a matrix of $\boldsymbol{x}_1, \cdots, \boldsymbol{x}_N$ elements, and $\boldsymbol{Z}$ be a matrix of $\boldsymbol{z}_1, \cdots, \boldsymbol{z}_N$. Then, their joint distribution is given by

$$p(\boldsymbol{X}, \boldsymbol{Z} | \boldsymbol{\theta}, \beta) = K^{-N} \prod_{n=1}^{N} \prod_{k=1}^{K} N(\boldsymbol{x}_n | \boldsymbol{y}_k, \beta^{-1}\boldsymbol{I})^{z_{Kn}}. \tag{1}$$

If the function $\boldsymbol{f}(\boldsymbol{u}_k; \boldsymbol{\theta})$ is a smooth nonlinear function, then nodes $\boldsymbol{u}_k$ are mapped onto $\boldsymbol{y}_k$ while maintaining the topological relationship in the latent space. GTM is seen as a mixture of Gaussian distributions, which means $\boldsymbol{y}_k$ are restricted to the lower dimensional manifold.

In GTM, the function $\boldsymbol{f}(\boldsymbol{u}_k; \boldsymbol{\theta})$ is constructed by a Gaussian process (GP) [3]. The nature of the GP is determined by the choice of a covariance function. The conventional GTM model uses a covariance function with a constant length scale throughout the latent space. This model cannot locally change the smoothness of the nonlinear function representing the distribution of the observed data according to the value of the latent variable. The underlying patterns of the element data are considered nonlinear and highly

complex, thus we require a GTM model that can represent more flexible functions. Therefore, we focused on GTM-LDLV [4], which is a recently proposed GTM model that can control the smoothness of the nonlinear function locally according to the value of the latent variable.

In GTM-LDLV, it is assumed that the D-dimensional feature vector $\boldsymbol{x}_n$ is generated independently by a restricted mixture of K Gaussian distributions defined in equation (1), and the nonlinear function $\boldsymbol{f}(\boldsymbol{u}_k)$ is modelled to be the product of two functions: a D-dimensional vector-valued function $\boldsymbol{h}(\boldsymbol{u}_k)$ and a positive scalar function $g(\boldsymbol{u}_k)$. Then, their joint distribution is given by

$$p(\boldsymbol{X}, \boldsymbol{Z}|\boldsymbol{g}, \boldsymbol{H}, \beta) = K^{-N} \prod_{n=1}^{N} \prod_{k=1}^{K} N(\boldsymbol{x}_n|\boldsymbol{y}_k, \beta^{-1}\boldsymbol{I})^{z_{Kn}}, \qquad \boldsymbol{y}_k = \boldsymbol{f}(\boldsymbol{u}_k) = g(\boldsymbol{u}_k)\boldsymbol{h}(\boldsymbol{u}_k),$$

where $\boldsymbol{g}$ is a vector $g(\boldsymbol{u}_k)\ (k = 1, \cdots, K)$, and $\boldsymbol{H}$ is a matrix $\boldsymbol{h}(\boldsymbol{u}_k)\ (k = 1, \cdots, K)$. The prior distribution of $g(\boldsymbol{u})$ is given as a truncated GP with mean 0 and covariance function $c_g(\boldsymbol{u}_i, \boldsymbol{u}_j; \boldsymbol{\xi}_g)$, which handles positive-bounded random functions. The prior distribution of the dth entry $h_d(\boldsymbol{u})$ of $\boldsymbol{h}(\boldsymbol{u})$ is given as a GP with mean 0 and covariance function $c_h(\boldsymbol{u}_i, \boldsymbol{u}_j)$. The prior distribution of the parameters g and $\boldsymbol{H}$ are given by

$$p(\boldsymbol{g}) = N^{+}\left(\boldsymbol{g}\middle|\boldsymbol{0}, \boldsymbol{C}_g(\boldsymbol{\xi}_g)\right), \tag{2}$$

$$p(\boldsymbol{H}|r) = \prod_{d=1}^{D} N\left(\boldsymbol{h}_{(d)}|\boldsymbol{0}, \boldsymbol{C}_h\right), \tag{3}$$

where N^{+} is a truncated normal distribution which handles positive-bounded random functions, $\boldsymbol{h}_{(d)}$ is a vector of the dth entry of the matrix $\boldsymbol{H}'$, and $\boldsymbol{C}_h$ is a matrix which consists of covariance function $c_h(\boldsymbol{u}_i, \boldsymbol{u}_j)$ as an element. Specifically, the covariance functions, $c_g(\boldsymbol{u}_i, \boldsymbol{u}_j; \boldsymbol{\xi}_g)$ and $c_h(\boldsymbol{u}_i, \boldsymbol{u}_j)$, are given by

$$c_g(\boldsymbol{u}_i, \boldsymbol{u}_j; \boldsymbol{\xi}_g) = v_g \cdot \exp\left(-\frac{\|\boldsymbol{u}_i - \boldsymbol{u}_j\|^2}{2l_g}\right), \tag{4}$$

$$c_h(\boldsymbol{u}_i, \boldsymbol{u}_j) = \left\{\frac{2l(\boldsymbol{u}_i)l(\boldsymbol{u}_j)}{l^2(\boldsymbol{u}_i) + l^2(\boldsymbol{u}_j)}\right\}^{\frac{L}{2}} \exp\left(-\frac{\|\boldsymbol{u}_i - \boldsymbol{u}_j\|^2}{l^2(\boldsymbol{u}_i) + l^2(\boldsymbol{u}_j)}\right). \tag{5}$$

In equation (4), the hyperparameter $\boldsymbol{\xi}_g$ consists of v_g and l_g, referred to as the variance and the length-scale respectively, that control the magnitude of variances and smoothness of a positive-valued function $g(\boldsymbol{u})$ generated from the GP. In equation (5), the length-scale parameter $l(\boldsymbol{u})$ is a function of $\boldsymbol{u}$ and parameterized as $l(\boldsymbol{u}) = \exp(r(\boldsymbol{u}))$ with the function $r(\boldsymbol{u})$ following GP with mean 0 and covariance function $c_r(\boldsymbol{u}_i, \boldsymbol{u}_j; \boldsymbol{\xi}_r)$. Finally, the prior distribution of the precision parameter β is given by

$$p(\beta) = \mathrm{Gam}(\beta|d_{\beta 0}, s_{\beta 0}), \tag{6}$$

where $\mathrm{Gam}(\cdot\,|d,s)$ denotes the gamma distribution, and its density function is defined by

$$\mathrm{Gam}(x|d,s)=\frac{s^d}{\Gamma(d)}x^{d-1}\exp(-sx),$$

where Γ is the gamma function $\Gamma(x)=\int_0^\infty e^{-t}t^{x-1}\,dt$.

The unknown parameter to be estimated is $\boldsymbol{\theta}=\{\boldsymbol{Z},\beta,\boldsymbol{g},\boldsymbol{H},\boldsymbol{r}\}$. In GTM-LDLV, the posterior distribution $p(\boldsymbol{\theta}|\boldsymbol{X})$ is approximately evaluated using a Markov Chain Monte Carlo (MCMC) method. Iteratively sampling from the full conditional posterior distribution for each member of $\{\boldsymbol{Z},\beta,\boldsymbol{g},\boldsymbol{H},\boldsymbol{r}\}$, we obtain a set of ensembles that follow the posterior distribution approximately. By taking the ensemble average over the samples from $p(\boldsymbol{\theta}|\boldsymbol{X})$, the parameters of GTM-LDLV are estimated. The simultaneous distribution of the data $\boldsymbol{X}$ and parameters $\boldsymbol{\theta}$ is given by

$$p(\boldsymbol{X},\boldsymbol{\theta})=p(\boldsymbol{X},\boldsymbol{Z}|\boldsymbol{g},\boldsymbol{H},\beta)p(\beta)p(\boldsymbol{g})p(\boldsymbol{H}|\boldsymbol{r})p(\boldsymbol{r}). \tag{7}$$

From equation (7) and Bayesian theorem, the posterior distribution of the latent variable $\boldsymbol{Z}$ is given by

$$p(\boldsymbol{Z}|\boldsymbol{X},\boldsymbol{\theta}_{-\boldsymbol{Z}})\propto p(\boldsymbol{X},\boldsymbol{\theta})\propto p(\boldsymbol{X},\boldsymbol{Z}|\boldsymbol{g},\boldsymbol{H},\beta)\propto\prod_{n=1}^{N}\prod_{k=1}^{K}\exp\left(-\frac{\beta}{2}\|\boldsymbol{x}_n-\boldsymbol{y}_k\|^2\right)^{z_{kn}}, \tag{8}$$

where $\boldsymbol{\theta}_{-\boldsymbol{A}}$ represents a set of the parameters obtained by removing $\boldsymbol{A}$ from $\boldsymbol{\theta}$. Since summation over k of $\boldsymbol{Z}$ for each n is equal to 1, equation (8) can be written as

$$p(\boldsymbol{Z}|\boldsymbol{X},\boldsymbol{\theta}_{-\boldsymbol{Z}})=\prod_{n=1}^{N}\prod_{k=1}^{K}\gamma_k(\boldsymbol{x}_n;\boldsymbol{g},\boldsymbol{H},\beta)^{z_{kn}}, \tag{9}$$

where $\gamma_k(\boldsymbol{x}_n)$ is the probability that $\boldsymbol{x}_n$ is generated by the kth mixing element given $\boldsymbol{X}$ and $\boldsymbol{\theta}_{-\boldsymbol{Z}}$. $\gamma_k(\boldsymbol{x}_n)$ is given by

$$\gamma_k(\boldsymbol{x}_n;\boldsymbol{g},\boldsymbol{H},\beta)=\frac{\exp\left(-\frac{\beta}{2}\|\boldsymbol{x}_n-\boldsymbol{y}_k\|^2\right)}{\sum_{k'=1}^{K}\exp\left(-\frac{\beta}{2}\|\boldsymbol{x}_n-\boldsymbol{y}_{k'}\|^2\right)}. \tag{10}$$

Next, from equation (10) and Bayesian theorem, the conditional posterior distribution for parameters $\beta,\boldsymbol{g},\boldsymbol{H}$, is given by

$$p(\beta|\boldsymbol{X},\boldsymbol{\theta}_{-\beta})=\mathrm{Gam}(\beta|d_\beta,s_\beta), \tag{11}$$

$$p(\boldsymbol{g}|\boldsymbol{X},\boldsymbol{\theta}_{-\boldsymbol{g}})=N^+(\boldsymbol{g}|\boldsymbol{\mu}_g,\boldsymbol{\Sigma}_g), \tag{12}$$

$$p(\boldsymbol{H}|\boldsymbol{X},\boldsymbol{\theta}_{-\boldsymbol{H}})=\prod_{d=1}^{D}N(\boldsymbol{h}_{(d)}|\boldsymbol{\mu}_{h,d},\boldsymbol{\Sigma}_h). \tag{13}$$

The parameters of the conditional posterior distribution for parameters $\beta, \boldsymbol{g}, \boldsymbol{H}$ are given by

$$d_\beta = d_{\beta 0} + \frac{ND}{2},$$

$$s_\beta = s_{\beta 0} + \frac{1}{2}\sum_{n=1}^{N}\sum_{k=1}^{K} z_{kn}\|\boldsymbol{x}_n - \boldsymbol{y}_k\|^2,$$

$$\boldsymbol{\mu}_g = \beta\boldsymbol{\Sigma}_g \mathrm{diag}(\boldsymbol{Z}\boldsymbol{X}'\boldsymbol{H}),$$

$$\boldsymbol{\Sigma}_g = (\beta\boldsymbol{G}\boldsymbol{\Lambda}_h + \boldsymbol{C}_{st}(\boldsymbol{\xi}_g)^{-1})^{-1},$$

$$\boldsymbol{\mu}_{h,d} = \beta\boldsymbol{\Sigma}_h\boldsymbol{\Lambda}_g\boldsymbol{Z}\boldsymbol{x}_{(d)}, \qquad \boldsymbol{\Sigma}_h = (\beta\boldsymbol{G}\boldsymbol{\Lambda}_g^2 + \boldsymbol{C}_h^{-1})^{-1}.$$

The posterior distribution of $\boldsymbol{r}$ is given by

$$p(\boldsymbol{r}|\boldsymbol{X}, \boldsymbol{\theta}_{-r}) \propto p(\boldsymbol{X}, \boldsymbol{\theta}) \propto p(\boldsymbol{H}|\boldsymbol{r})p(\boldsymbol{r}) \propto \exp\big(s(\boldsymbol{r})\big),$$

$$s(\boldsymbol{r}) = -\frac{D}{2}\ln|\boldsymbol{C}_h| - \frac{1}{2}\sum_{d=1}^{D}\boldsymbol{h}'_{(d)}\boldsymbol{C}_h^{-1}\boldsymbol{h}_{(d)} - \frac{1}{2}\boldsymbol{r}'\boldsymbol{C}_{st}(\xi_r)^{-1}\boldsymbol{r}. \tag{14}$$

Since $\boldsymbol{C}_h$ is a matrix that depends on $\boldsymbol{r}$, a sampling of $\boldsymbol{r}$ is performed as follows using Metropolis-Hasting method [5]. First, find the local maximum point $\hat{\boldsymbol{r}}$ of the log-likelihood function $s(\boldsymbol{r})$, then generate the candidate point $\boldsymbol{r}^*$ from the proposed distribution $N(\boldsymbol{r}|\boldsymbol{m}_r, \boldsymbol{V}_r)$. $\boldsymbol{m}_r, \boldsymbol{V}_r$ are given by

$$\boldsymbol{m}_r = \hat{\boldsymbol{r}} + \boldsymbol{V}_r \frac{\partial s(\boldsymbol{r})}{\partial \boldsymbol{r}}\bigg|_{\boldsymbol{r}=\hat{\boldsymbol{r}}}, \qquad \boldsymbol{V}_r = \left\{-\frac{\partial^2 s(\boldsymbol{r})}{\partial\boldsymbol{r}\partial\boldsymbol{r}'}\right\}^{-1}_{\boldsymbol{r}=\hat{\boldsymbol{r}}}.$$

When the current point is $\boldsymbol{r}^{t-1}$, the candidate point $\boldsymbol{r}^*$ is accepted with the next probability.

$$\min\left\{\frac{\exp\big(s(\boldsymbol{r}^*)\big)\,N(\boldsymbol{r}^{t-1}|\boldsymbol{m}_l, \boldsymbol{V}_l)}{\exp\big(s(\boldsymbol{r}^{t-1})\big)\,N(\boldsymbol{r}^*|\boldsymbol{m}_l, \boldsymbol{V}_l)}, 1\right\}. \tag{15}$$

Algorithm of PTG

The algorithm of PTG is summarized in Algorithm 1.

Algorithm 1 Periodic Table Generator (PTG)

1: Prepare initial value $\boldsymbol{\theta}^0 = \{\boldsymbol{Z}^0, \beta^0, \boldsymbol{g}^0, \boldsymbol{H}^0, \boldsymbol{r}^0\}$.

for $t = 1$ to T **do**

Sample $\boldsymbol{Z}^t$ from $p(\boldsymbol{Z}|\boldsymbol{X}, \beta^{t-1}, \boldsymbol{g}^{t-1}, \boldsymbol{H}^{t-1}, \boldsymbol{r}^{t-1})$.

Sample β^t from $p(\beta|\boldsymbol{X}, \boldsymbol{Z}^t, \boldsymbol{g}^{t-1}, \boldsymbol{H}^{t-1}, \boldsymbol{r}^{t-1})$.

Sample $\boldsymbol{g}^t$ from $p(\boldsymbol{g}|\boldsymbol{X}, \boldsymbol{Z}^t, \beta^t, \boldsymbol{H}^{t-1}, \boldsymbol{r}^{t-1})$.

Sample $\boldsymbol{H}^t$ from $p(\boldsymbol{H}|\boldsymbol{X}, \boldsymbol{Z}^t, \beta^t, \boldsymbol{g}^t, \boldsymbol{r}^{t-1})$.

Sample $\boldsymbol{r}^t$ from $p(\boldsymbol{r}|\boldsymbol{X}, \boldsymbol{Z}^t, \beta^t, \boldsymbol{g}^t, \boldsymbol{H}^t)$.

end for

For a sufficiently large number T_b, record $\boldsymbol{\theta}^t = \{\boldsymbol{Z}^t, \beta^t, \boldsymbol{g}^t, \boldsymbol{H}^t, \boldsymbol{r}^t\}, t = T_b, T_b + 1, \cdots, T$.

2: The model parameters of GTM-LDLV $\boldsymbol{\theta}^{ldlv} = \{\boldsymbol{Z}^{ldlv}, \beta^{ldlv}, \boldsymbol{g}^{ldlv}, \boldsymbol{H}^{ldlv}, \boldsymbol{r}^{ldlv}\}$ are estimated by taking the average of $\boldsymbol{\theta}^t = \{\boldsymbol{Z}^t, \beta^t, \boldsymbol{g}^t, \boldsymbol{H}^t, \boldsymbol{r}^t\}$ for $t = T_b, T_b + 1, \cdots, T$. Increase the number of nodes on the latent space so that $K \geq N$ is satisfied. Considering the parameters estimated by GTM-LDLV (The first step of PTG) as observation values, interpolate the parameters corresponding to the newly generated nodes using GP regression.

3: The parameters $\boldsymbol{\theta}^{itp} = \{\boldsymbol{Z}^{itp}, \beta^{itp}, \boldsymbol{g}^{itp}, \boldsymbol{H}^{itp}, \boldsymbol{r}^{itp}\}$ obtained as above are used as initial values for the next procedure.

for $t = 1$ to T' **do**

$\boldsymbol{Z}^t \leftarrow \underset{\boldsymbol{Z} \in A}{\operatorname{argmax}}\, p(\boldsymbol{Z}|\boldsymbol{X}, \beta^{t-1}, \boldsymbol{g}^{t-1}, \boldsymbol{H}^{t-1}, \boldsymbol{r}^{t-1}),\ A = \{\boldsymbol{Z} | \sum_{n=1}^{N} z_{kn} \leq 1\ (k = 1, \cdots, K)\}$.

$\beta^t \leftarrow \underset{\beta}{\operatorname{argmax}}\, p(\beta|\boldsymbol{X}, \boldsymbol{Z}^t, \boldsymbol{g}^{t-1}, \boldsymbol{H}^{t-1}, \boldsymbol{r}^{t-1})$.

$\boldsymbol{g}^t \leftarrow \underset{\boldsymbol{g}}{\operatorname{argmax}}\, p(\boldsymbol{g}|\boldsymbol{X}, \boldsymbol{Z}^t, \beta^t, \boldsymbol{H}^{t-1}, \boldsymbol{r}^{t-1})$.

$\boldsymbol{H}^t \leftarrow \underset{\boldsymbol{H}}{\operatorname{argmax}}\, p(\boldsymbol{H}|\boldsymbol{X}, \boldsymbol{Z}^t, \beta^t, \boldsymbol{g}^t, \boldsymbol{r}^{t-1})$.

$\boldsymbol{r}^t \leftarrow \underset{\boldsymbol{r}}{\operatorname{argmax}}\, p(\boldsymbol{r}|\boldsymbol{X}, \boldsymbol{Z}^t, \beta^t, \boldsymbol{g}^t, \boldsymbol{H}^t)$.

end for

Notes on the PTG Algorithm

It should be noted that PTG may produce different visualization results for each trial even under the same hyper parameter settings. Indeed, PTG with the element data produced different tables for each trial of the algorithm. This implies that PTG reached around different local maxima on the likelihood surface for each trial. PTG tries to fit lower dimensional manifolds to the shape of data cloud, and there should be multiple solutions to this. Therefore, it is expected that there are many local maxima which are separated from one other on the likelihood surface of PTG. This is not counterintuitive as there should not be a unique optimal solution for arranging elements in the new periodic table. One way to deal with this problem is to run the algorithm multiple times under the same hyperparameter settings and enumerate multiple visualization results. The final result is then selected from the list of the obtained tables based on some selection criterion.

In Step 1 of PTG with the elements' data, it was observed that the learning of the model became unstable and was terminated when the non-information prior distribution was used as prior distribution of the precision β. To address the problem, a prior distribution of β with a small scale and a sufficiently large rate was used. This prior distribution keeps the variance β^{-1} estimated from the posterior distribution larger than a certain value, and it made the learning stable.

In the next section, we introduce details of the analysis procedure and hyper parameter settings used in this study.

Details of Analysis Procedure

We performed PTG on two different node layouts namely, square and three-dimensional conical layouts. In the square layout of $L = 2$, we set $K = 25$ in the first step of PTG in which the 5×5 nodes were evenly arranged on the area $[-1, 1] \times [-1, 1]$. In the second step, we increased the number of nodes to 9×9 by placing new nodes at middle points on the line segments connecting between each node. In the conical layout of $L = 3$, we first used a set of nodes with $K = 25$ that were arranged uniformly on the surface of the cone placed in the area $[-1, 1] \times [-1, 1] \times [-1, 1]$. The cone was sliced into 4 sections of the same height along the vertical axis. Then, 1 (vertex), 4, 8, and 12 (bottom) nodes were uniformly placed on the outer part of the 4 cut surfaces. In the following step, the number of slices was increased by 7, and 1 (vertex), 4, 8, 12, 16, 20, and 24 (bottom) nodes were uniformly arranged in the same way. In

both cases, we set $\xi_g = \xi_r = (1/3,3)$, the number of iteration in MCMC was set to $T = 10{,}000$ with the burn-in step $T_b = 5{,}000$, the number of iteration in the third step of fine-tuning was set to $T = 10$, and PTG was run 10 times under the same hyper parameter settings written above.

To quantitatively evaluate the quality of the periodic tables obtained by PTG with the same hyper parameter settings and different trials, we considered using a table as an element descriptor in machine learning tasks. The modelling procedure and the data set that was used is the same as the one written in the section of 'Quantitative comparison of periodic tables'. We performed the five-fold cross-validation on the 12,373 samples for the obtained 10 periodic tables. The prediction errors for the 10 periodic tables are shown in Fig. S6 for the square table and Fig. S7 for the conical table. As shown in Fig. S6, the 10th square periodic table gave the lowest MAE (0.533 eV/atom) out of 10 tables. Therefore, this table was chosen as the final visualization result of the square PTG table, and it corresponds to that shown in Fig. 2. Similarly, as shown in Fig. S7, the 4th conical periodic table giving the lowest MAE (0.464 eV/atom) was chosen as the final visualization result of the conical PTG table, and it corresponds to that shown in Fig. 3.

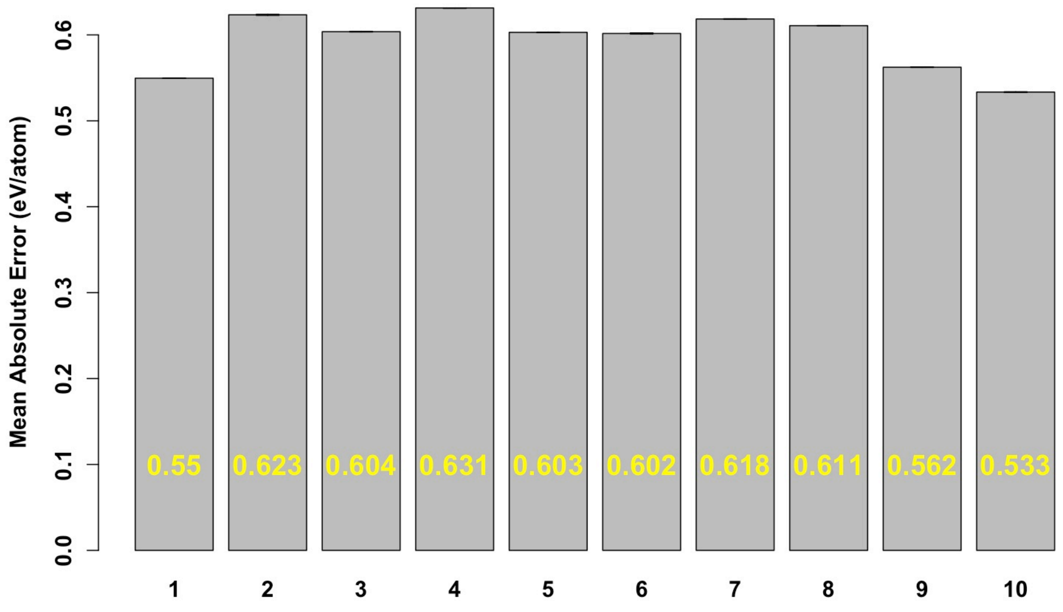

Figure S6. Mean absolute errors (MAE) of the prediction of the formation energy per atom for the 10 square periodic tables used as element descriptors. The vertical axis indicates cross-validated MAE of

random forest regressors (RF) trained with the 10 descriptors obtained from the coordinates of elements in the square periodic tables produced by PTG, with the same hyper parameters and different trials. The error bars denote the standard deviations in 5 independent trials of the cross-validation.

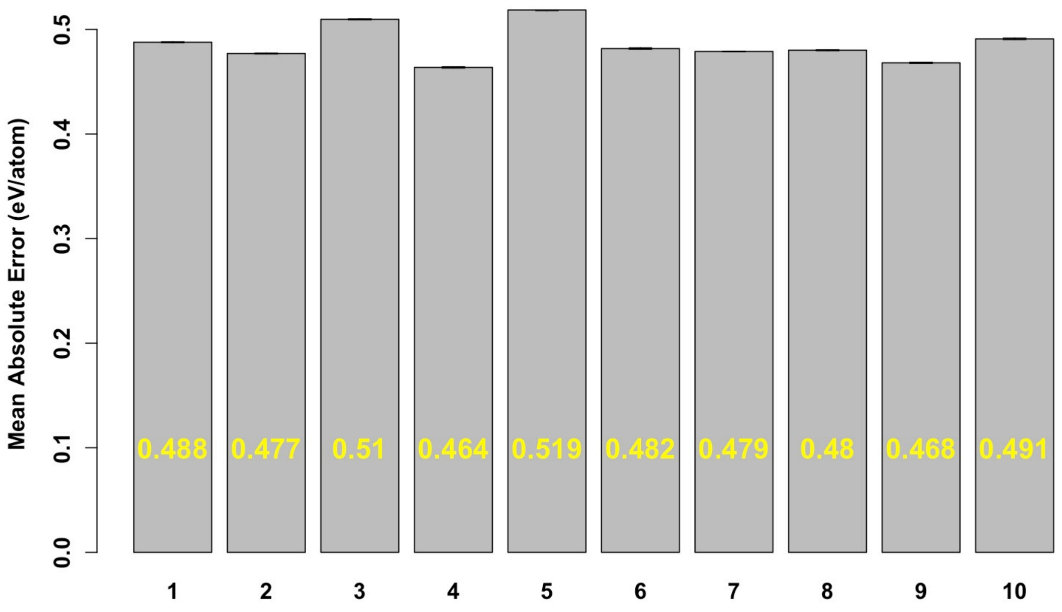

Figure S7. Mean absolute errors (MAE) of the prediction of the formation energy per atom for the 10 conical periodic tables used as element descriptors. The vertical axis indicates cross-validated MAE of random forest regressors (RF) trained with the 10 descriptors obtained from the coordinates of elements in the conical periodic tables produced by PTG, with the same hyper parameters and different trials. The error bars denote the standard deviations in 5 independent trials of the cross-validation.

Other Examples

Additionally, we performed the PTG on another three different layouts of nodes: rectangle, three-dimensional cylinder, and cubic layouts. In the rectangle layout of $L = 2$, we set $K = 27$ in the first step in which the 3×9 nodes were evenly arranged on the area $[-1, 1] \times [-1, 1]$. In the second step, we increased the number of nodes to 5×17 by placing new nodes at the middle points of the line segments connecting between each node. Then finally, in order to have the same layout as the standard periodic table (5×18), we added a column of 5 nodes in the

positive direction of the x-axis. In the cylinder layout of $L = 3$, we first used a set of nodes with $K = 24$ that were arranged uniformly on the surface of the cylinder placed in the area $[-1, 1] \times [-1, 1] \times [-1, 1]$. The cylinder was sliced into 3 sections in the same height along the vertical axis. Then, 8 nodes were uniformly placed on the outer part of the 3 cut surfaces. In the next step, the number of slices was increased by 5, and 16 nodes were uniformly arranged in the same way. In the cubic layout of $L = 3$, we set $K = 27$ in the first step in which the $3 \times 3 \times 3$ nodes were evenly arranged on the area $[-1, 1] \times [-1, 1] \times [-1, 1]$. In the second step, we increased the number of nodes to $5 \times 5 \times 5$ by placing new nodes at the middle points of the line segments connecting between each node. In all the three cases, the element data, the conditions of hyper-parameters, and the analysis procedure are completely the same to the square and conical cases. The results are shown in Fig. S8.

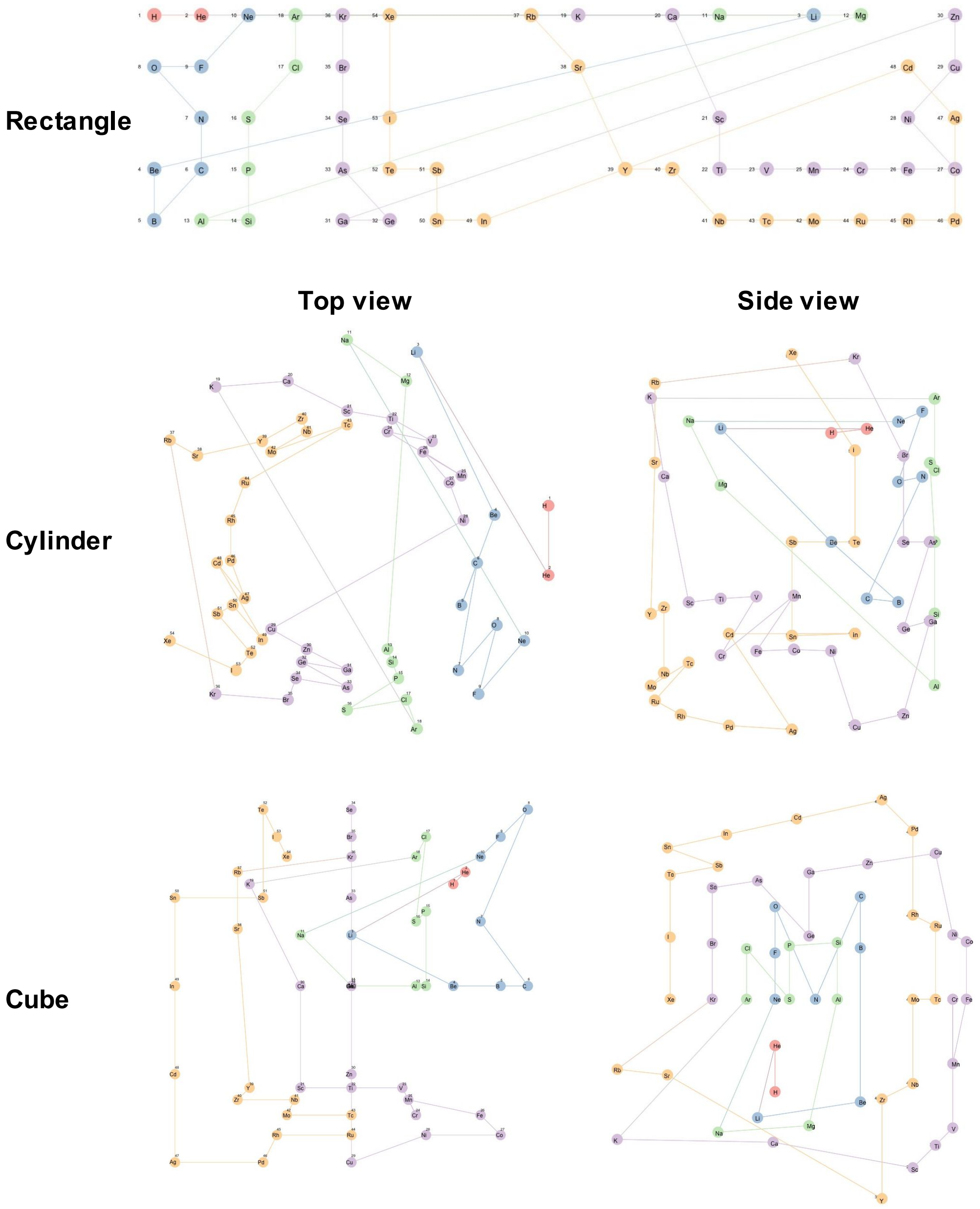

Figure S8. Examples of the PTG tables with three different layouts: rectangular grids (top), three-dimensional cylinder type (middle), and cubic (bottom). The elements are colour-coded according to the five periods and numbered by atomic numbers. A line passing through the elements is drawn in the order of the atomic numbers. For the cylinder and the cubic tables, the left and right figures show the views from top and side, respectively.

References

[1] Bishop, C. M., Svensén, M. & Williams, C. K. I. GTM: the generative topographic mapping. *Neural Computation.* **10**, 215-234 (1998).

[2] Kohonen, T. Self-organized formation of topologically correct feature maps. *Biological Cybernetics.* **43**, 59-69 (1982).

[3] Williams, C. K. I. Prediction with Gaussian process: from linear regression to linear prediction and beyond. *Learning and Inference in Graphical Models*. 599-621 (1997).

[4] Yamaguchi, N. GTM with latent variable dependent length-scale and variance. *CACS.* DOI: https://doi.org/10.1109/CACS.2013.6734192 (2013).

[5] Hasings, W. K. Monte Carlo sampling methods using Markov chains and their applications. *Biometrika*. 97-109 (1970).
